# A resorcin[4]arene hexameric capsule as a supramolecular catalyst in elimination and isomerization reactions

**DOI:** 10.3762/bjoc.18.38

**Published:** 2022-03-28

**Authors:** Tommaso Lorenzetto, Fabrizio Fabris, Alessandro Scarso

**Affiliations:** 1 Dipartimento di Scienze Molecolari e Nanosistemi, Università Ca’ Foscari di Venezia, via Torino 155, 30172, Mestre-Venezia, Italy

**Keywords:** cationic intermediates, encapsulation, organocatalysis, resorcin[4]arene hexamer, supramolecular catalysis

## Abstract

The hexameric resorcin[4]arene capsule as a self-assembled organocatalyst promotes a series of reactions like the carbonyl–ene cyclization of *(S)-*citronellal preferentially to isopulegol, the water elimination from 1,1-diphenylethanol, the isomerization of α-pinene and β-pinene preferentially to limonene and minor amounts of camphene. The role of the supramolecular catalyst consists in promoting the protonation of the substrates leading to the formation of cationic intermediates that are stabilized within the cavity with consequent peculiar features in terms of acceleration and product selectivity. In all cases the catalytic activity displayed by the hexameric capsule is remarkable if compared to many other strong Brønsted or Lewis acids.

## Introduction

**Scheme 1:**
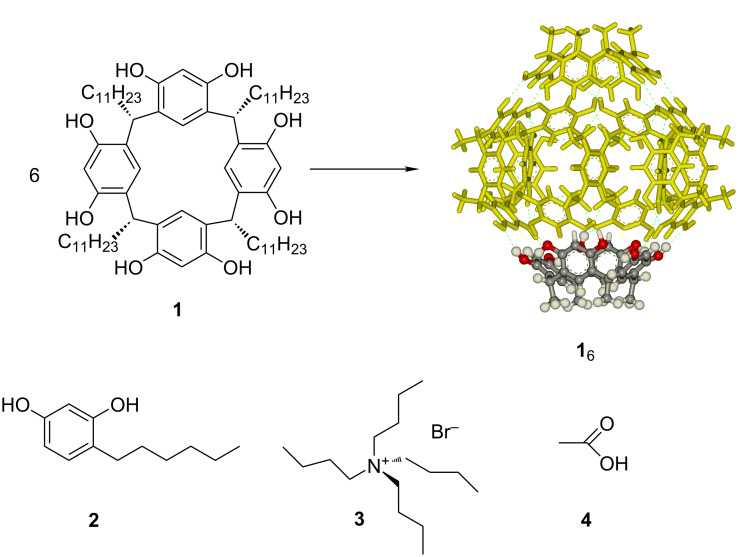
Resorcin[4]arene **1** forming the corresponding hexameric capsule **1**_6_ and the species used for control experiments like 4-*n*-hexylresorcinol (**2**) to mimic the H-bonding properties of the capsule, tetrabutylammonium bromide (**3**) to act as a competitive guest for the cavity and acetic acid (**4**) to mimic just the Brønsted acidity of the capsule.

In enzymatic catalysis, the substrate is selected matching the size, shape and specific functional groups present in the active site of the enzyme. Once bound, substrate activation is carried out by specific amino acid side chains that adorn the inner surface of the cavity by means of a combination of covalent and/or weak intermolecular interactions leading to the stabilization of intermediate species and transition states of the reaction with impressive accelerations, substrate, and product selectivities. The development of artificial catalytic systems able to activate substrates and to stabilize intermediate species through weak noncovalent interactions is the scope of supramolecular catalysis [1–4]. Several examples of supramolecular catalysts [5–7] have been introduced in the recent years [3,8] where the catalytic host is designed to bind substrates, accelerate reactions, and steer product [9–12] and substrate [13–14] selectivities thanks to confinement effects [15–17]. Many host structures endowed with catalytic features are full covalent units (e.g., cyclodextrins, cucurbiturils, pillararenes and other derivatives) whose synthesis can be sometimes time consuming. Alternatively, supramolecular catalysts can be obtained through self-assembling units [18–19], metal-ligand components [20] or hydrogen bonding units. In the latter case, a key role is played by the resorcin[4]arene **1** as a one-step multigram synthesis product, that spontaneously self-assembles in wet apolar solvents like chloroform or benzene forming a hexameric structure [21] thanks to the formation of a seam of sixty hydrogen bonds between six units of **1** and eight water molecules (Scheme 1). A very recent publication investigated different possible assemblies that are present in solution as function of the water content [22]. The pseudo-spherical host structure is highly dynamic, with a large cavity of about 1375 Å^3^ that is accessible [23] for cationic guests [24] thanks to extended cation–π interactions [25] as well as other electron poor molecules [26–27]. After our seminal work on the use of **1**_6_ as a nanoreactor to bind an Au(I) catalyst and to impart unique product [28] as well as substrate [29] selectivity, the capsule attracted the attention of several other research groups bringing this self-assembled nanocontainer to the forefront in the field of supramolecular catalysts. The specific field of research has been recently reviewed [19,30–33] to underline the potentialities of this simple and efficient organocatalyst. Recent promoted reactions by the resorcin[4]arene capsule include the intramolecular ether cyclization [34], the synthesis of bis(heteroaryl)methanes [35], the imine formation [36], the Michael addition reactions of *N*-methylpyrrole on methyl vinyl ketone [37], the synthesis of sesquiterpene natural product derivatives [38–39] and the carbonyl olefin metathesis leading to 2,5-dihydropyrroles [40].

Herein we present our investigation on the ability of the hexameric capsule **1**_6_ to act as a supramolecular self-assembled organocatalyst for a series of unimolecular reactions involving the formation of cationic intermediate species [41] like water elimination from an alcohol to provide the corresponding alkene, the isomerization of β-pinene and α-pinene and the cyclization of (*S*)*-*citronellal to secondary alcohols. The key point to interpret the action of the capsule in all these reactions is the combination of its weak Brønsted acidity [42] that, by protonation of the substrate, leads to the formation of cationic species [43] and the stabilization of the latter through cation–π interactions [44] within the electron-rich aromatic cavity of the capsule, thus providing acceleration of the reaction and specific product selectivities.

## Results and Discussion

We decided to explore the application of the resorcin[4]arene capsule **1**_6_ to new classes of unimolecular reactions which are known to occur through the formation of cationic intermediate species, that can be expected to be efficiently stabilized within the electron-rich cavity of the capsule. Aiming at ascertaining the real role played by the self-assembled capsule, the solvent chloroform-*d* was previously passed on basic alumina in order to avoid traces of HCl, that recently demonstrated to combine catalytic effects with the nano-environment provided by the capsule [45]. Moreover, all reactions were also tested i) using 4-*n*-hexylresorcinol (**2**) as a molecular unit able to provide the same hydrogen bonding properties of the resorcin[4]arene but lacking the formation of the cavity ii) in the presence of the capsule with an excess of tetrabutylammonium bromide (**3**) as a competitive guest for the capsule, to demonstrate the importance of the presence of an accessible cavity, and iii) acetic acid (**4**) in order to mimic only the Brønsted acidity of the capsule without providing the stabilization properties related to the presence of electron-rich aromatic surfaces.

In a recent publication, Reek [22] further investigated the self-assembly properties of **1** as function of the content of water in chloroform-*d* demonstrating that the symmetrical hexameric capsule **1**_6_·(H_2_O)_8_ comprising 8 water molecules (capsule A) is present only with water content lower than 50 mM, while in the presence of increasing amounts of water a second capsule B is also present comprising overall 15 water molecules, 6−7 of which spontaneously incorporated into a single edge of the cubic suprastructure leading to increased Brønsted acidity. Therefore, we performed extra control experiments for each investigated reaction also only with the more symmetrical and less acidic capsule A to underline the effect of the water content on the catalytic activity.

### (*S*)-Citronellal isomerization to cyclic secondary alcohols

**Scheme 2:**
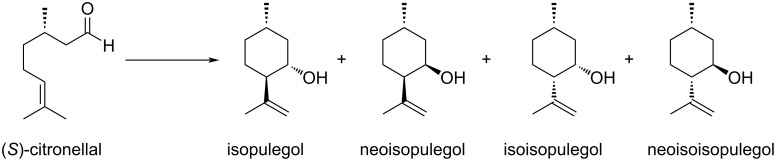
Carbonyl–ene intramolecular cyclization of (*S*)-citronellal to the corresponding diastereoisomeric cyclic secondary alcohols.

(*S*)*-*Citronellal is an enantiopure monoterpenoid, isolated from oils distilled from citronella plants, which is very effective as a repellent and antifungal. In the presence of Brønsted or Lewis acids (*S*)-citronellal undergoes an intramolecular carbonyl–ene cyclization reaction forming two new stereogenic centers, which turns out into four possible diastereoisomeric secondary alcohols (Scheme 2).

**Figure 1:**
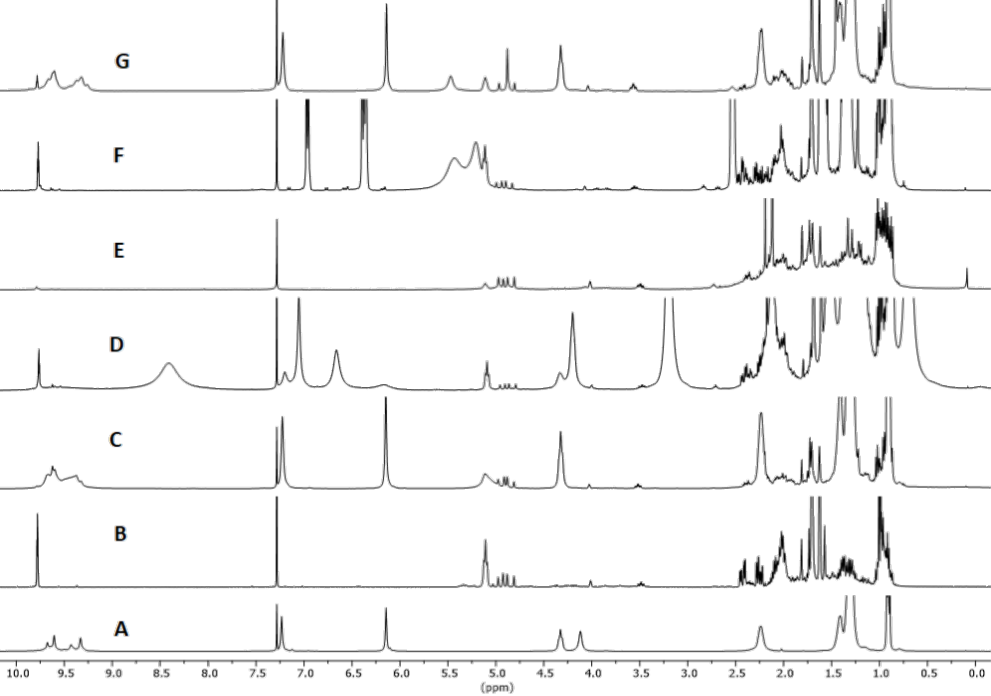
^1^H NMR spectra in water-saturated CDCl_3_ except for G. A: [**1**_6_] (7.5 mM); B: citronellal; C: citronellal (75 mM), [**1**_6_] (7.5 mM); D: citronellal (75 mM), [**1**_6_] (7.5 mM), Bu_4_NBr (**3**, 78 mM); E: citronellal (75 mM), acetic acid (29 mM); F: citronellal (75 mM), *n*-hexylresorcinol (**2**, 30 mM); G: citronellal (75 mM), [**1**_6_·8H_2_O] (7.5 mM). Spectra recorded after 24 h at 60 °C, while A and B were recorder right after sample preparation.

The reaction of (*S*)-citronellal in the presence of 10 mol % of **1**_6_ at 60 °C was monitored by ^1^H NMR observing the rapid disappearance of the triplet signal at 9.78 ppm relative to the aldehyde hydrogen atom and consequent increase of the signals at 4.9 ppm relative to the vinylic hydrogen atoms of the isopulegol obtained in higher amount with respect to neoisopulegol (Figure 1 and Table 1, entry 1). In order to achieve larger substrate conversion, the reaction was repeated for 72 h at 60 °C observing almost complete substrate conversion and high isopulegol to neoisopulegol ratio (Table 1, entry 2). The reaction with **1**_6_ occupied by **3** as a competitive guest led to a marked decrease of catalytic activity, and negligible conversion was observed using 4-*n*-hexylresorcinol (**2**) as a molecule with similar H-boding properties compared to **1** (Figure 1D and E; Table 1, entries 3 and 4). The reaction promoted by acetic acid (**4**) as purely Brønsted acid led to comparable conversion of the substrate with respect to the use of **1**_6_, albeit with much similar product distribution between isopulegol and neoisopulegol, even extending the reaction time up to 72 h at 60 °C (Table 1, entries 5 and 6). The reaction with the symmetrical capsule A (**1**_6_·8H_2_O) characterized by lower intrinsic acidity [22] led to lower conversion with respect to the same reaction in the presence of high water content (Figure 1G; Table 1, entry 7). Overall for this reaction the capsule acts primarily as a Brønsted acid and the presence of the accessible cavity of the capsule steers product distribution.

**Table 1:** Conversion and selectivity for the carbonyl–ene isomerization of (*S*)-citronellal at 60 °C for 24 h.

#	Catalyst	Conv. (%)	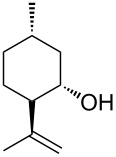 Isopulegol (%)	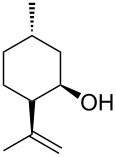 Neoisopulegol (%)

1	**1**_6_^a^	49	32	15
2	**1**_6_^a ,b^	95	73	22
3	**1**_6_ + **3**	0	0	0
4	**2**	6	3	3
5	AcOH (**4**)^a^	49	21	26
6	AcOH (**4**)^a,b^	60	28	32
7	capsule A **(1**_6_·8H_2_O)^a^	33	23	8

^a^3% of other diastereoisomeric secondary alcohols; ^b^60 °C for 72 h.

It is worth to note that the preferred product isopulegol is an important intermediate product in the industrial production of menthol by the Takasago and BASF processes [46–47]. Many catalytic methods for the cyclization of citronellal have been developed mostly based on transition metals, both as heterogeneous and homogeneous catalysts frequently under much harsher experimental conditions [48–50]. The same cyclization of citronellal was reported also by Raymond and collaborators in water using a tetrahedral polyanionic metal-ligand capsule as supramolecular catalyst, observing lower catalytic activity and product distribution favoring isopulegol but also with large amounts of neoisopulegol and neoisoisopulegol (52%, 32% and 11%, respectively) [51]. More favorable selectivity towards isopulegol was obtained by the same group with a larger tetrahedral capsule using pyrene in place of naphthalene units observing isopulegol, neoisopulegol and neoisoisopulegol with 71%, 24% and 4% selectivity [52]. The comparison between the metal-ligand tetrahedral capsules and the H-bonded **1**_6_ shows a common trend of selectivity towards isopulegol with increasing the volume of the cavity.

As a further investigation concerning other possible isomerization reactions involving protonation of the substrate, and formation of possible cationic intermediate species, we tested the menthone isomerization to isomenthone [53] without observing any product formation with 10 mol % of capsule after 24 h at 60 °C. Similarly, the carvone isomerization [54] involving protonation of the exocyclic double bond of the substrate followed by two hydride shifts and aromatization to carvacrol did not proceed at all with 10 mol % of capsule after 24 h at 60 °C. Carvone isomerization was achieved at 60 °C for 24 h only combining the use of 10 mol % of capsule and 10 mol % of HBF_4_. Nevertheless, the reaction under identical conditions, but in the presence of 10 equiv of **3** as competitive guest, led only to a minimal inhibition of the reaction, thus excluding a fundamental role of the cavity under such experimental conditions for carvone isomerization.

### Dehydration of 1,1-diphenylethanol to 1,1-diphenylethylene

**Scheme 3:**
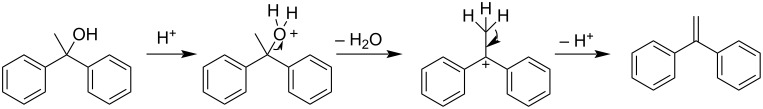
Dehydration reaction of 1,1-diphenylethanol to 1,1-diphenylethylene.

The dehydration reaction of 1,1-diphenylethanol to 1,1-diphenylethylene occurs with protonation at the oxygen atom of the reagent with subsequent water elimination leading to a stable tertiary, bis-benzyl carbocation that evolves forming the corresponding alkene by proton elimination (Scheme 3). The reaction was carried out at 60 °C with 10 mol % of **1**_6_ (Table 2, entry 1), monitoring the formation of the products by ^1^H NMR and GC–MS (see Supporting Information File 1).

**Table 2:** Dehydration of 1,1-diphenylethanol to 1,1-diphenylethylene 3 h and 20 h at 60 °C.

#	Catalyst	Yield (%, 3 h)	Yield (%, 20 h)

1	**1**_6_	87	100
2	**1**_6_ + **3**	0	3
3	**2**	0	2
4	AcOH (**4**)	0	1
5	capsule A (**1**_6_·8H_2_O)	44	100

**Figure 2:**
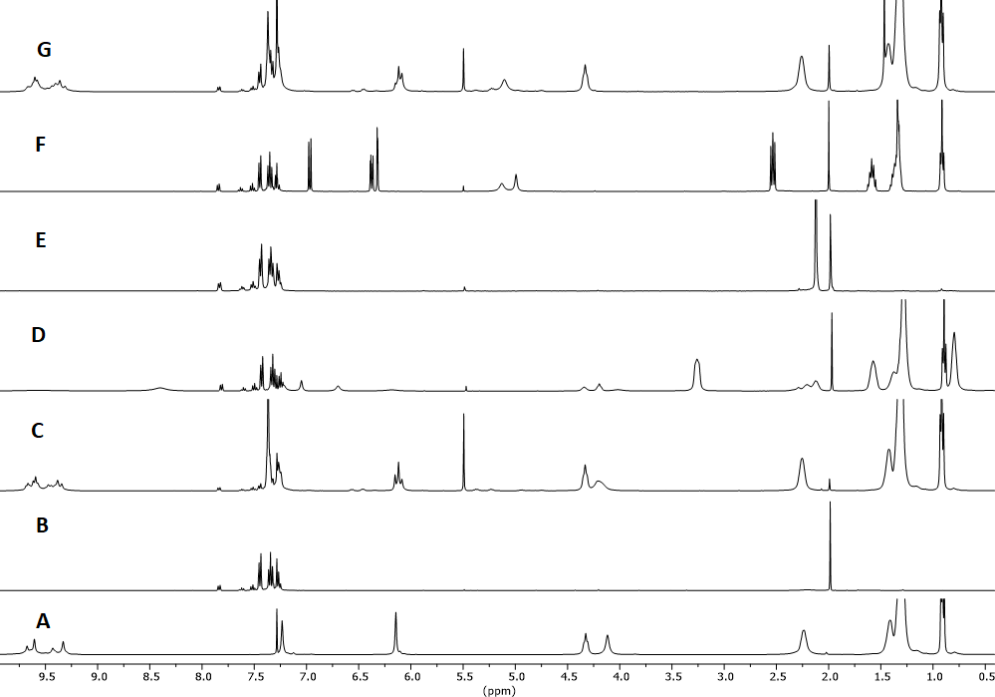
^1^H NMR spectra in water-saturated CDCl_3_ except for G. A: [**1**_6_] (7.5 mM); B: 1,1-diphenylethanol; C: 1,1-diphenylethanol (75 mM), [**1**_6_] (7.5 mM); D: 1,1-diphenylethanol (75 mM), [**1**_6_] (7.5 mM), Bu_4_NBr (**3**, 78 mM); E: 1,1-diphenylethanol (75 mM), acetic acid (29 mM); F: 1,1-diphenylethanol (75 mM), *n*-hexylresorcinol (**2**, 30 mM); G: 1,1-diphenylethanol (75 mM), [**1**_6_·8H_2_O] (7.5 mM). Spectra recorded after 3 h at 60 °C, while A and B were recorder right after sample preparation.

From the ^1^H NMR spectra it was possible to follow the consumption of the substrate characterized by a singlet at 2.00 ppm attributed to the methyl hydrogens and the appearance of the characteristic singlet signal of the vinyl hydrogens at 5.5 ppm for the product (Figure 2), with quantitative yield after 20 h at 60 °C in the presence of 10 mol % of the capsule. The same reaction carried out with the capsule **1**_6_ and in the presence of **3** as competitive guest led to complete inhibition of the formation of the corresponding alkene (Figure 2D, Table 2, entry 2). This provides evidence of the importance of an accessible cavity for the activation of the reaction after alcohol protonation. It is known that **1**_6_ behaves as a weakly acidic assembly with p*K*_a_ of about 5.5 [42], while the resorcinol moiety presents a p*K*_a_ of 9.15. This enhanced acidity is likely to promote the protonation of the substrate. The reaction was repeated with **2** as a hydrogen bonding unit and with acetic acid (**4**) as a comparable Brønsted acid observing in both cases that the formation of 1,1-diphenylethylene was negligible (Figure 2E and F, Table 2, entries 3 and 4). Further control experiments with the capsule A (**1**_6_·8H_2_O) with water content lower than 50 mM showed after 3 h much lower product formation with respect to the same reaction with higher water content (Figure 2G and Table 2, entry 5), in agreement with the lower acidity of capsule A [22] that leads to lower formation of the intermediate carbocation species. It has also to be considered that water elimination during the progress of the reaction changes the ratio between capsules A and B thus accelerating product formation. The intrinsic stability of the carbocation formed by water elimination from the corresponding alcohol is crucial to observe activation of the reaction by the capsule. In fact, the reaction with the capsule under identical experimental conditions using 1-phenylethanol as substrate did not provide any evidence of formation of the corresponding alkene product even after 20 h at 60 °C. Secondary aliphatic alcohols can be efficiently dehydrated by the capsule only in combination with HCl, leading to an acceleration of two orders of magnitude with respect to the same reaction in the absence of the supramolecular catalyst [55]. The group of Tiefenbacher reported about the reactivity of tertiary alcohols bearing a trisubstituted alkene in the side chain [56] for which the reaction catalyzed by the same capsule led to the protonation of the alkene unit with an intramolecular nucleophilic attack by the alcohol moiety forming a final cyclic ether product. In the latter case the tertiary alcohol could not easily eliminate water and protonation occurred preferentially on the alkene moiety.

It is worth to note that the present dehydration reaction of 1,1-diphenylethanol with **1**_6_ occurs under mild experimental conditions with respect to recent examples in the literature operating with strong Brønsted and Lewis acids like sulfuric acid [57], *p*-toluenesulfonic acid [58–59] or with scandium tris(trifluoromethanesulfonate) [60]. These results clearly speak for the combined effect provided by the capsule consisting in sufficient Brønsted acidity and concomitant stabilization of the cationic intermediate within the electron-rich cavity leading to the promotion of the elimination reaction.

### β-Pinene isomerization

**Scheme 4:**
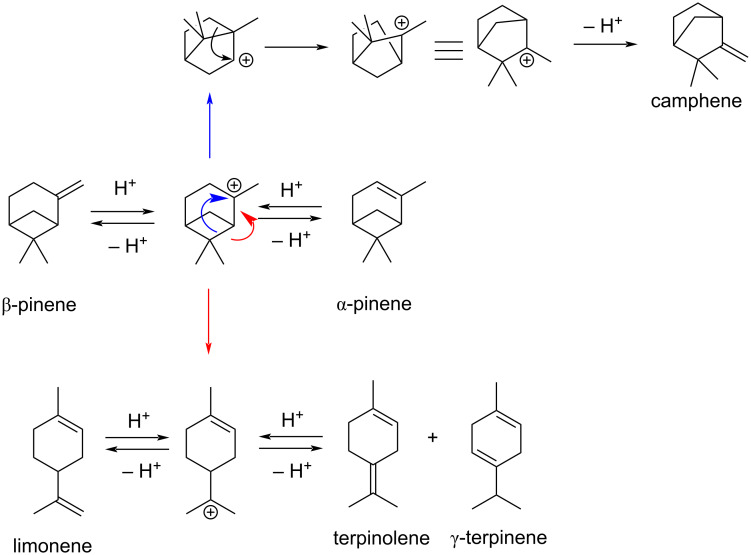
Possible isomerization products from β-pinene and α-pinene.

β-Pinene is a bicyclic monoterpene bearing an exocyclic C=C double bond that can be recovered in large quantities from the production processes of cellulose from the wood of conifers. β-Pinene is in equilibrium with the more stable endocyclic alkene α-pinene by protonation. From the common cationic intermediate species, many rearrangement products can be obtained, some of which derived by the ring opening of the tensioned 4-membered ring (Scheme 4).

**Figure 3:**
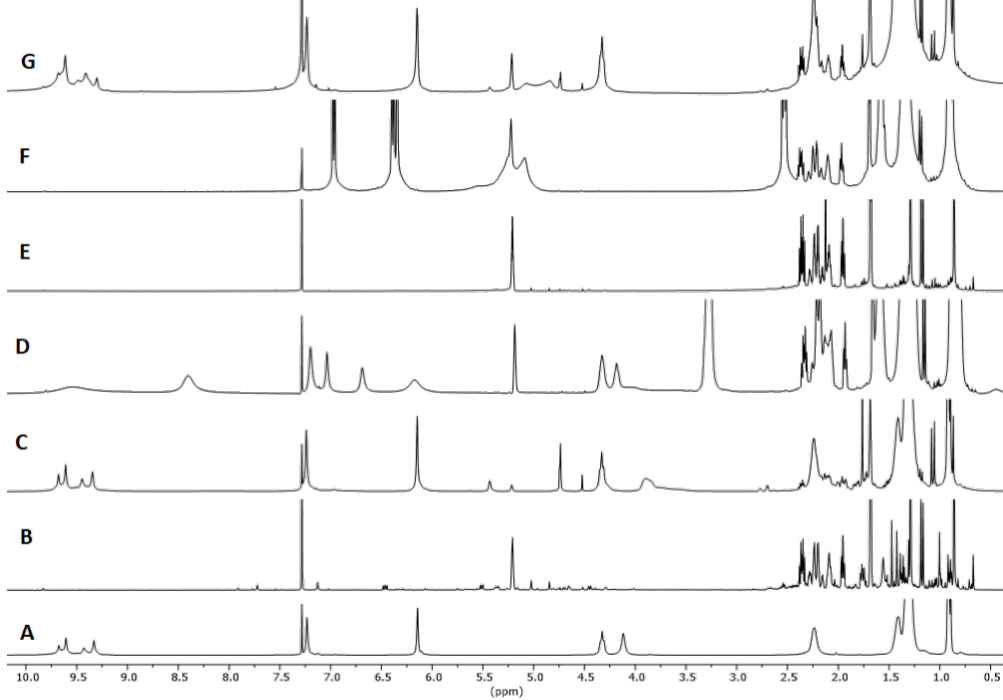
^1^H NMR spectra in water-saturated CDCl_3_ except for G. A: [**1**_6_] (7.5 mM); B: α-pinene; C: α-pinene (75 mM), [**1**_6_] (7.5 mM); D: α-pinene (75 mM), [**1**_6_] (7.5 mM), Bu_4_NBr (**3**, 78 mM); E: α-pinene (75 mM), acetic acid (29 mM); F: α-pinene (75 mM), *n*-hexylresorcinol (**2**, 30 mM); G: α-pinene (75 mM), [**1**_6_·8H_2_O] (7.5 mM). Spectra recorded after 24 h at 60 °C, while A and B were recorder right after sample preparation.

α-Pinene possesses a more stable endocyclic double bond and therefore tends to react less rapidly than β-pinene. The reaction of α-pinene was monitored following the disappearing of the resonance of the vinylic hydrogen atom at 5.23 ppm. Concomitant formation of a new resonance at 5.43 attributed to limonene and at 4.52 for camphene was observed. In the presence of 10 mol % of **1**_6_ substrate conversion after 24 h at 60 °C corresponded to 75%, leading to double amounts of limonene with respect to camphene (Figure 3C and Table 3 entries 1 and 2). All the control experiments with the cavity of the capsule occupied by the competitive guest **3**, or with **2** or acetic acid as catalysts led to no product formation under identical experimental conditions (Figure 3D, E and F, Table 3, entries 3–5).

**Table 3:** Product distribution for the isomerization reaction of α-pinene at 60 °C.

#	Catalyst	Time	Conv. (%)	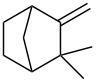 Camphene (%)	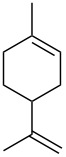 Limonene (%)

1	**1**_6_	3 h	5	3	2
2	**1**_6_	24 h	75^a^	24	50
3	**1**_6_ + **3**	24 h	0	–	–
4	**2**	24 h	0	–	–
5	AcOH (**4**)	24 h	0	–	–
6	capsule A (**1**_6_·8H_2_O)	3 h	3	2	1
7	capsule A (**1**_6_·8H_2_O)	24 h	15	5	10

^a^1% of other monocyclic isomerization products.

The reaction carried out with capsule A (**1**_6_·8H_2_O) obtained with low water content led to diminished catalytic activity with similar product distribution compared to the reaction containing both capsules (Figure 3G, Table 3, entries 6 and 7). As suggested in the literature [22], the higher Brønsted acidity of the capsule B present under high water content could also in this case be responsible for the larger formation of the cationic intermediate further stabilized within the capsule with consequent enhanced catalytic activity.

**Figure 4:**
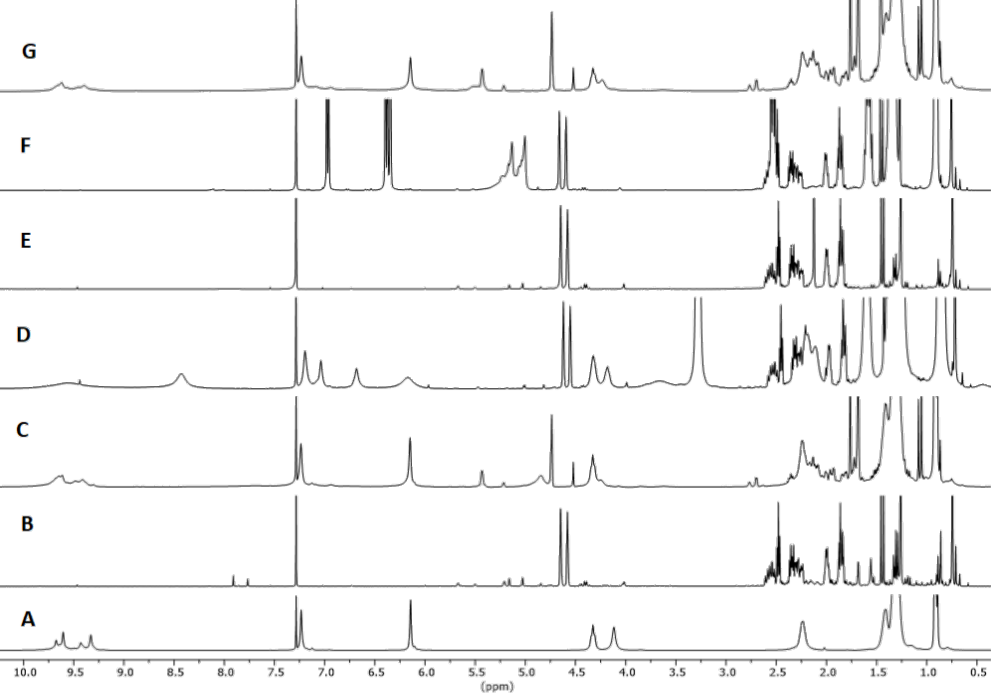
^1^H NMR spectra in water-saturated CDCl_3_ except for G. A: [**1**_6_] (7.5 mM); B: β-pinene; C: β-pinene (75 mM), [**1**_6_] (7.5 mM); D: β-pinene (75 mM), [**1**_6_] (7.5 mM), Bu_4_NBr (**3**, 78 mM); E: β-pinene (75 mM), acetic acid (29 mM); F: β-pinene (75 mM), *n*-hexylresorcinol (**2**, 30 mM); G: β-pinene (75 mM), [**1**_6_·8H_2_O] (7.5 mM). Spectra recorded after 24 h at 60 °C, while A and B were recorder right after sample preparation.

β-Pinene is characterized by a more reactive, exocyclic double bond and this led to higher conversions compared to α-pinene (Table 4 and Figure 4). By ^1^H NMR it was observed the decrease in intensity of the signal at 4.60 ppm characteristic for the vinylic H atoms of β-pinene and the appearance of different signals related to the formation of limonene as major product, camphene, α-pinene and other monocyclic isomerization products like terpinolene, α- and γ-terpinene, in traces (Table 4, entries 1, 2 and 3). Quantitative conversion was observed with 10 mol % of **1**_6_ after 24 h at 60 °C, while the reaction with **1**_6_ in the presence of 10 equiv of the competitive ammonium guest **3** led to a drastic reduction of the catalytic activity (Table 4, entries 3 and 4). No conversion was detected in the presence of 4-*n*-hexylresorcinol (**2**), and the same was observed for the reaction in the presence of acetic acid (**4**, Table 4, entries 5 and 6, respectively). The reaction with capsule A (**1**_6_·8H_2_O) [22] showed a higher conversion to products at 1.5 and 3 h reaction compared to capsule **1_6_** (see Supporting Information File 1), with almost identical product yields after 24 h. The faster activity of capsule A (**1**_6_·8H_2_O) with respect to **1**_6_ observed with β-pinene is opposite to what observed for α-pinene, and clearly cannot be related to the specific acidities of the two capsules A and B.

**Table 4:** Product distribution for the isomerization reaction of β-pinene at 60 °C.

#	Catalyst	Time	Conv. (%)	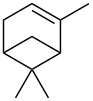 α-Pinene (%)	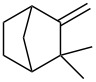 Camphene (%)	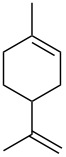 Limonene (%)

1	**1**_6_	1.5 h	14	2	4	8
2	**1**_6_	3 h	30	4	9	17
3	**1**_6_	24 h	100^a^	10	26	62
4	**1**_6_ + **3**	24 h	0	–	–	–
5	**2**	24 h	0	–	–	–
6	AcOH (**4**)	24 h	0	–	–	–
7	capsule A (**1**_6_·8H_2_O)	1.5 h	35	5	10	20
8	capsule A (**1**_6_·8H_2_O)	3 h	62	8	18	36
9	capsule A (**1**_6_·8H_2_O)	24 h	100^a^	1	30	67

^a^2% of other monocyclic isomerization products.

**Figure 5:**
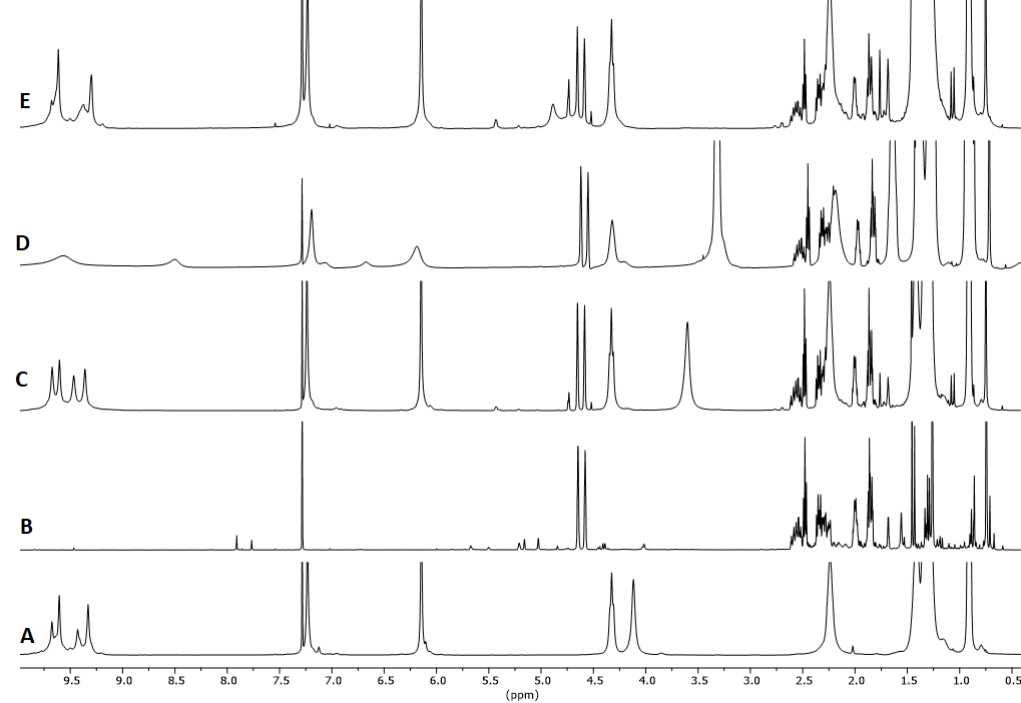
^1^H NMR spectra in water-saturated CDCl_3_, except for E. A: [**1**_6_] (7.5 mM); B: β-pinene; C: β-pinene (75 mM), [**1**_6_] (7.5 mM); D: β-pinene (75 mM), [**1**_6_] (7.5 mM), Bu_4_NBr (**3**, 78 mM); E: β-pinene (75 mM), [**1**_6_^.^8H_2_O] (7.5 mM). Spectra recorded after 64 h at room temperature, while A and B were recorder right after sample preparation.

Due to the complete conversion observed at 60 °C, we also tested the isomerization of β-pinene at room temperature (Figure 5 and Table 5) confirming that substrate conversion was higher when using the symmetrical capsule A (**1**_6_·8H_2_O) under low water content conditions [22], with respect to capsule **1_6_** as mixture of A and B (Figure 5A and E). A possible explanation could be related to the fact that β-pinene is a higher energy isomer with respect to α-pinene and because of this it does not beneficiate much of the higher acidity of capsule B compared to capsule A. Both capsules provide sufficient protonation to β-pinene leading to the carbocationic intermediate species which is likely to be stabilized more effectively by the more symmetric and tighter capsule A (**1**_6_·8H_2_O), which is held on by the minimum amount of water to form the network of hydrogen bonds with hydroxy moieties. Indeed, the capsule B, is looser because of the presence of extra water molecules that can arguably interact with their hydrogens with the lone pairs of the hydroxy moieties of the aromatic units thus diminishing the electron density of the cavity, which results less stabilizing for the carbocationic intermediate.

**Table 5:** Product distribution for the isomerization reaction of β-pinene at rt for 64 h.

#	Catalyst	Conv. (%)	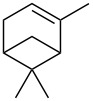 α-Pinene (%)	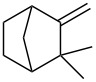 Camphene (%)	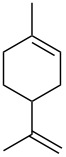 Limonene (%)

1	**1**_6_	8	1	3	4
2	**1**_6_ + **3**	0	–	–	–
3	capsule A (**1**_6_·8H_2_O)	20	3	6	11

All these experiments demonstrated that the reaction was promoted by **1**_6_. The Brønsted acidity of the capsule led to the formation of the intermediate carbocationic species that was stabilized within the cavity of the hexamer and converted to limonene as major product. We also tried to intercept the carbocationic intermediate using good nucleophiles in equimolar amounts such as *N*-methylindole or 2-mercaptoethanol but in all cases the reactions conducted in the presence of such nucleophiles proceeded forming only the isomerization products.

It is worth to notice that the conversion observed with the capsule was comparable to that what was frequently observed with typical heterogeneous Lewis or Brønsted catalysts under harsher experimental conditions with *T* higher than 100 °C in the gas phase [61]. More importantly, the product distribution observed is rather unusual if compared to many other heterogeneous catalytic systems known in the literature for leading to higher selectivity for camphene [62–66], and less likely to comparable amounts of camphene and limonene [67–68]. The preferential formation of limonene, which compared to camphene is a much more valuable product, has been less frequently observed, for instance in the case of heteropolyacids on SBA-15 [69]. The use of the capsule **1**_6_ represents a competitive catalytic system especially considering the milder experimental conditions, the lack of strong Brønsted or Lewis acids and the simple supramolecular catalytic approach.

## Conclusion

In conclusion, we described a series of examples of supramolecular catalysis where the hexameric capsule **1**_6_ acts mimicking enzymes by combination of the activation of substrates by protonation, stabilization of cationic intermediate species with consequent acceleration of its conversion and selective product formation thanks to weak intermolecular interactions between the cationic intermediates and the internal surface of the capsule. Specifically, the capsule efficiently promoted the carbonyl–ene cyclization of (*S*)-citronellal forming isopulegol as the main product, the elimination of water from 1,1-diphenylethanol forming 1,1-diphenylethylene, the isomerization of α-pinene and β-pinene forming preferentially limonene with respect to camphene. For these unimolecular reactions the use of the capsule represents a competitive catalytic system with respect to several literature methods especially considering the mild experimental conditions and absence of strong Brønsted or Lewis acids. A series of control experiments supported in all cases the evidence that the reaction is not just promoted by the acidity of **1**_6_ or its H-bonding properties, but that a combination of weak Brønsted acidity together with the presence of an accessible electron-rich cavity that favors the stabilization of the cationic intermediates is crucial for the supramolecular catalytic activity observed. In the reactions studied, the capsule plays a crucial role in steering product selectivity, thus further stimulating the investigation of new reactions.

## Experimental

### Instrumentation and operating conditions

The ^1^H NMR spectra were acquired at 298 K with a Bruker AVANCE 300 spectrometer operating at 300.15 MHz and with a Bruker 400 spectrometer operating at 400.15 MHz. The chemical shift values (δ) in ppm refer to SiMe_4_ (TMS) as standard. For GC–MS analysis a GC Trace GC 2000 apparatus was used equipped with an HP5-MS column (30 m, ID 0.25 mm, 0.5 μm film), the carrier gas used was He coupled to a quadrupole MS Thermo Finnigan Trace MS (full scan, EI at 70 eV).

All the chemicals are commercially available and were used as received by the seller without further purification. The water-saturated chloroform-*d* was prepared by adding, at room temperature, a few drops of bi-distilled water to a 100 mL bottle chloroform-*d* previously passed on basic alumina in agreement with recent literature results to avoid interference by HCl [45]. Resorcin[4]arene **1** was synthesized and purified according to the procedure reported in the literature [49,70–71].

### Experiments with substrates and capsule

**1** (0.027 mmol, 6 equiv with respect to the hexameric capsule, 45 mM, 10 mol % of capsule with respect to substrate) was dissolved in an NMR tube containing 0.6 mL of water-saturated chloroform-*d* previously passed on basic alumina. Subsequently, the substrate (75 mM) was introduced, and the tube was maintained at 60 °C. The progress of the reaction was monitored by ^1^H NMR and GC–MS by periodic sampling of the solution. Product assignments in the ^1^H NMR spectra were carried out in accordance with literature data: for citronellal isomerization products [72–75], for 1,1-diphenylethene [76], for pinenes isomerization products [77–78].

#### Control experiments with 4-*n*-hexylresorcinol (**2**)

4-*n*-Hexylresorcinol (**2**, 0.108 mmol, 30 mM, 24 equiv with respect to the capsule) was dissolved in an NMR tube containing 0.6 mL of water-saturated chloroform-*d* previously passed on basic alumina. Subsequently, the substrate was introduced (75 mM), and the tube was heated at 60 °C. The progress of the reaction was monitored by ^1^H NMR and GC–MS by periodic sampling of the solution.

#### Control experiments with capsule and competitive ammonium guests **3**

In an NMR tube containing 0.6 mL of water-saturated chloroform-*d* previously passed on basic alumina, **1** (0.027 mmol, 6 equiv with respect to the capsule, 45 mM) was dissolved. Tetrabutylammonium bromide (**3**, 10 equiv with respect to the capsule, 75 mM) was added and the tube heated until clear. Subsequently, the substrate was added (75 mM), and the tube maintained at 60 °C. The progress of the reaction was monitored by ^1^H NMR and GC–MS by periodic sampling of the solution.

#### Control experiments with acetic acid (**4**)

The substrate (75 mM) was introduced into an NMR tube with 0.6 mL of water-saturated chloroform-*d* previously passed on basic alumina. Subsequently, acetic acid (**4**, 30 mM, 4 equiv with respect to the capsule) was added. The tube was heated at 60 °C. The progress of the reaction was monitored by ^1^H NMR and GC–MS by periodic sampling of the solution.

#### Control experiments with low water content and hexameric capsule A **(1**_6_·8H_2_O)

Compound **1** (500 mg) was triturated with cyclohexane (10 mL) and the latter was removed under vacuum with gentle heating in order to remove the azeotropic mixture of water/cyclohexane. The treatment with cyclohexane was repeated two more times. The solid product was left under high vacuum for 20 h to remove traces of cyclohexane. A mother solution of **1** (0.27 mmol, 6 equiv with respect to the hexameric capsule, 45 mM) was prepared with 6 mL of chloroform-*d* previously passed on basic alumina which provide sufficiently dry solvent. To favor dissolution the mixture was heated gently and sonicated. This provided a solution of the hexameric capsule A (**1**_6_·8H_2_O) with water content <50 mM in agreement with a recent publication by Reek [22].

## Supporting Information

File 1 Details on experimental proceduresand ^1^H NMR spectra for the catalytic tests.
