# Supplementary material for: A resorcin[4]arene hexameric capsule as a supramolecular catalyst in elimination and isomerization reactions
Source: Beilstein J Org Chem. 2022 Mar 28;18:337–49. doi: 10.3762/bjoc.18.38 (PMC8978922; doi:10.3762/bjoc.18.38)
Supplement: File 1 — Details on experimental proceduresand 1H NMR spectra for the catalytic tests. [file Beilstein_J_Org_Chem-18-337-s001.pdf]

BEILSTEIN JOURNAL OF ORGANIC CHEMISTRY

Supporting Information

for

A resorcin[4]arene hexameric capsule as a supramolecular catalyst in elimination and isomerization reactions

Tommaso Lorenzetto, Fabrizio Fabris and Alessandro Scarso

Beilstein J. Org. Chem. **2022,** *18,* 337–349. doi:10.3762/bjoc.18.38

Details on experimental proceduresand ^{1}H NMR spectra for the catalytic tests

License and Terms: This is a supporting information file under the terms of the Creative Commons Attribution License (https://creativecommons.org/licenses/by/4.0). Please note that the reuse, redistribution and reproduction in particular requires that the author(s) and source are credited and that individual graphics may be subject to special legal provisions.
The license is subject to the *Beilstein Journal of Organic Chemistry* terms and conditions: (https://www.beilstein-journals.org/bjoc/terms)

Citronellal isomerization: experiment with capsule [$\mathbf{1}_6$]

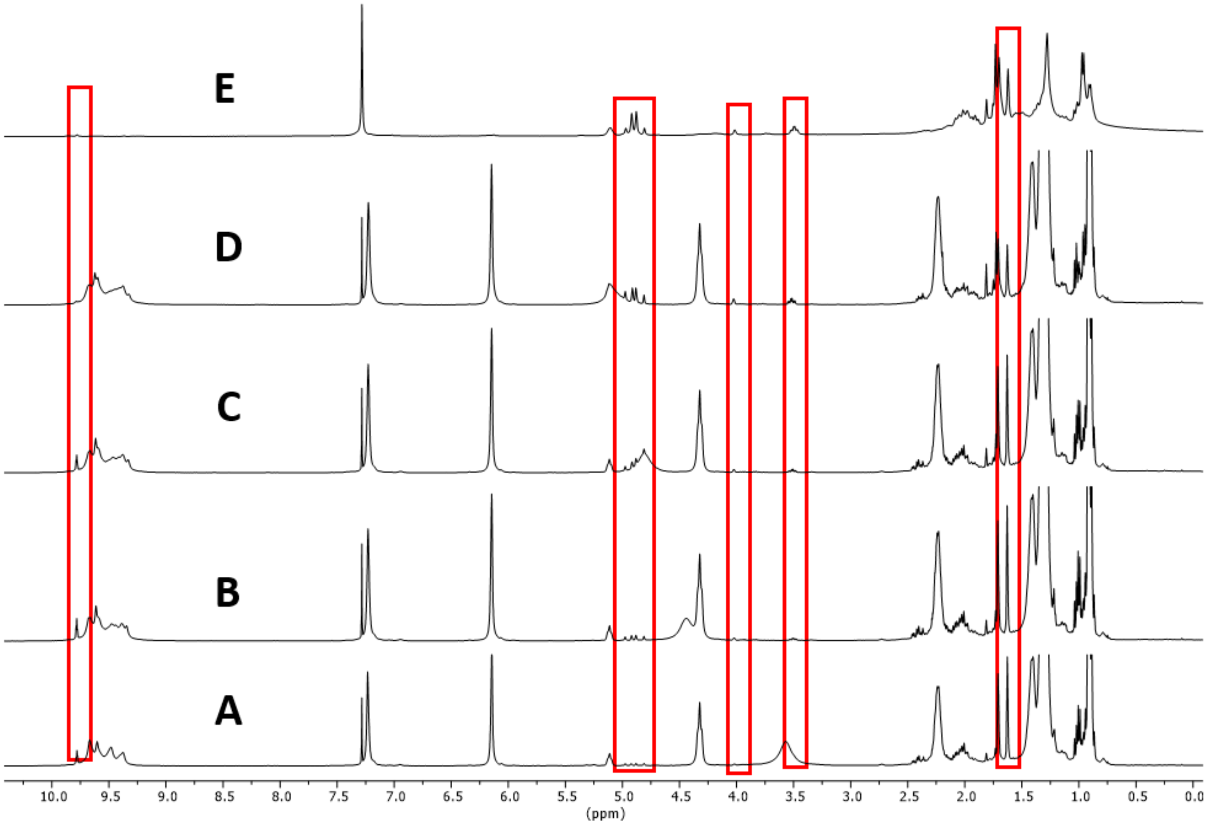

Figure S1: ^{1}H NMR spectra in water-saturated $CDCl_3$ of a solution of citronellal (75 mM), [$\mathbf{1}_6$] (7.5 mM), recorded after preparation (A), after 1.5 h (B), 3 h (C), 24 h (D), 72 h (E) at 60 °C.

Citronellal isomerization: control experiments with capsule [$\mathbf{1}_6$] and competitive ammonium guests **3**

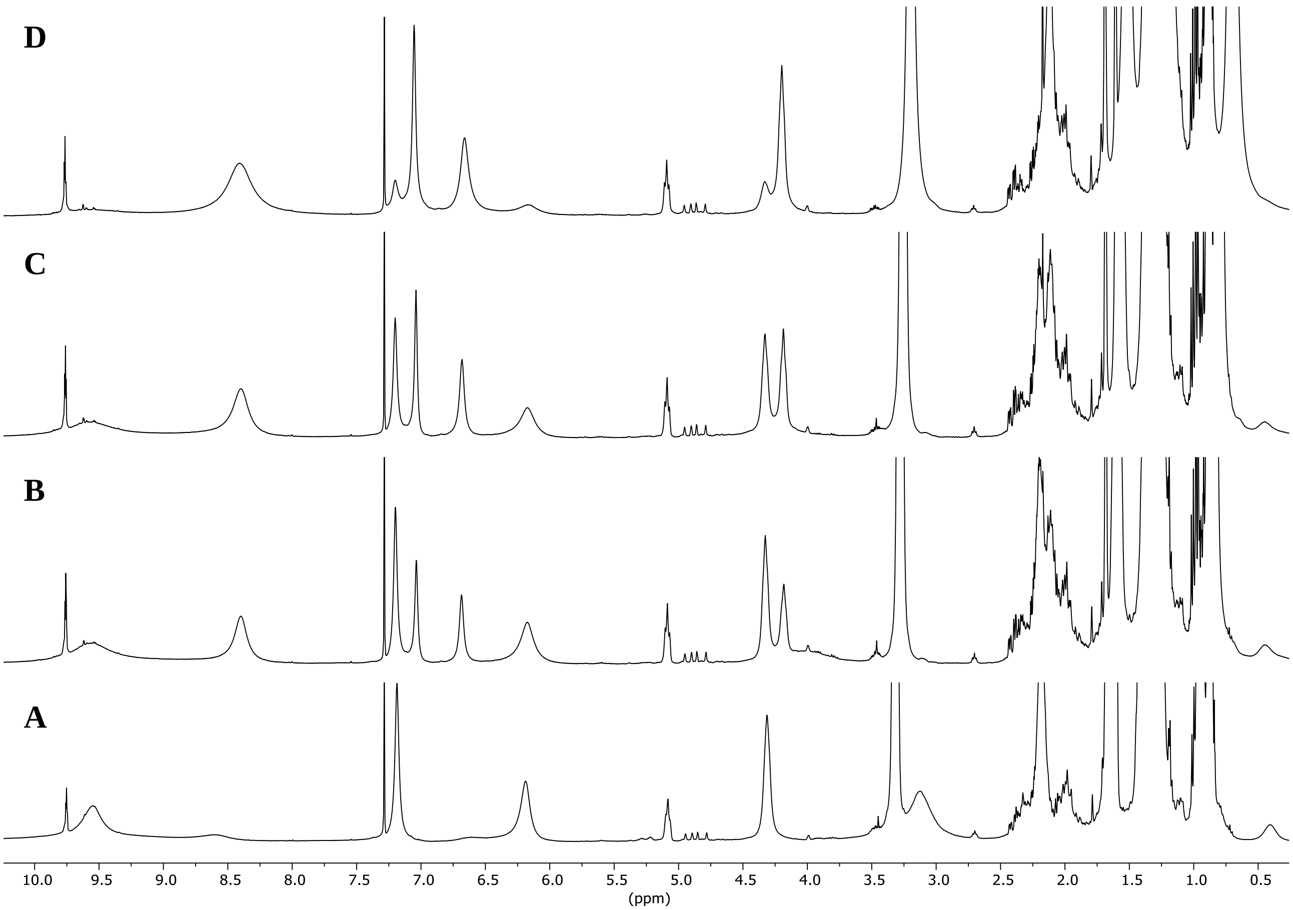

Figure S2: ^{1}H NMR spectra in water-saturated $CDCl_3$ of a solution of citronellal (75 mM), [$\mathbf{1}_6$] (7.5 mM), Bu_4NBr **3** (78 mM) recorded after preparation (A), after 1.5 h (B), 3 h (C), 24 h (D) at 60 °C.

<u>**Citronellal isomerization:** control experiments with 4-*n*-hexyl-resorcinol **2**</u>

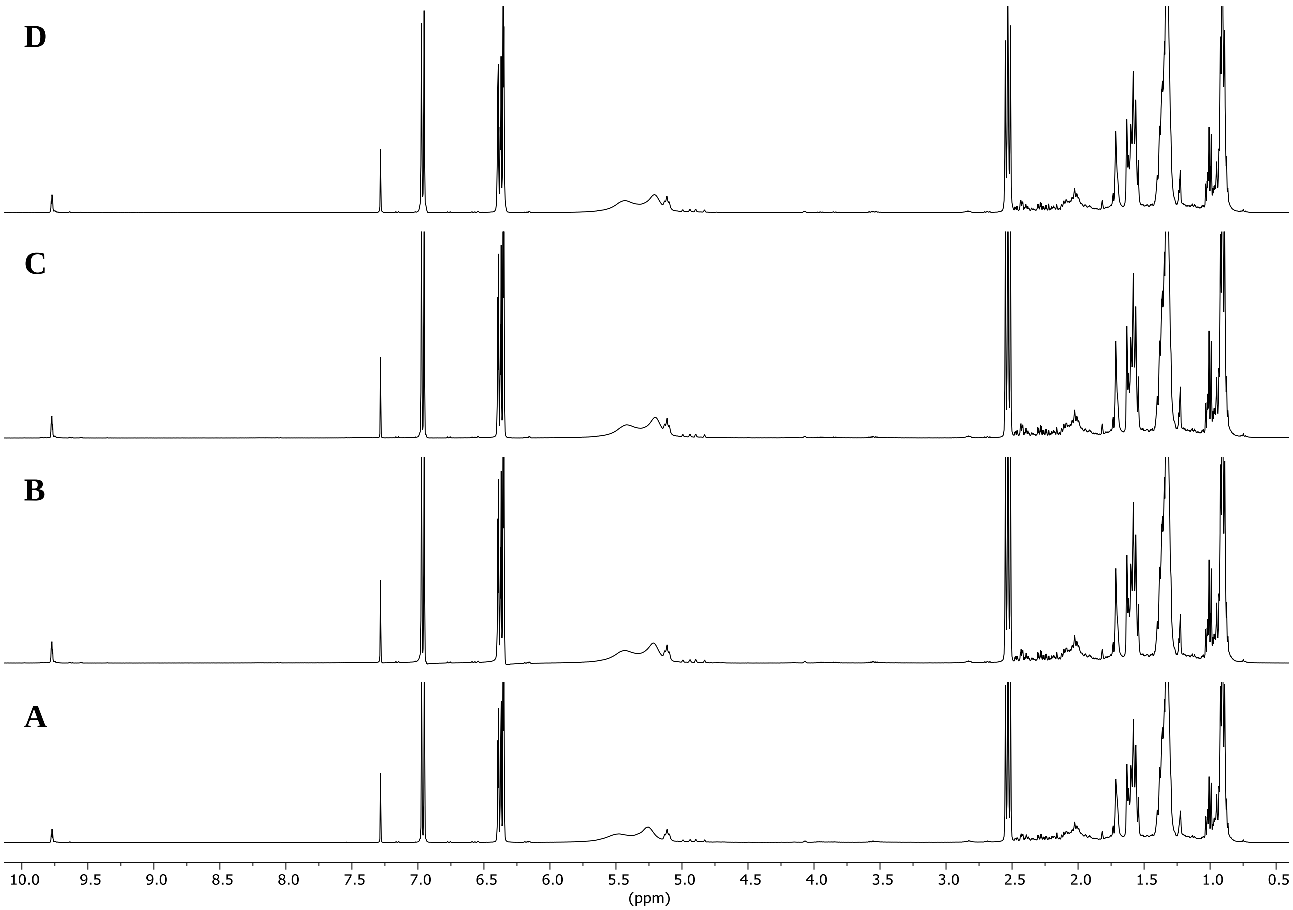

Figure S3:^{1}H NMR spectra in water-saturated $CDCl_3$ of a solution of citronellal (75 mM), n-hexylresorcinol **2** (30 mM) recorded after preparation (A), after 1.5 h (B), 3 h (C), 24 h (D) at 60 °C.

Citronellal isomerization: control experiments with acetic acid

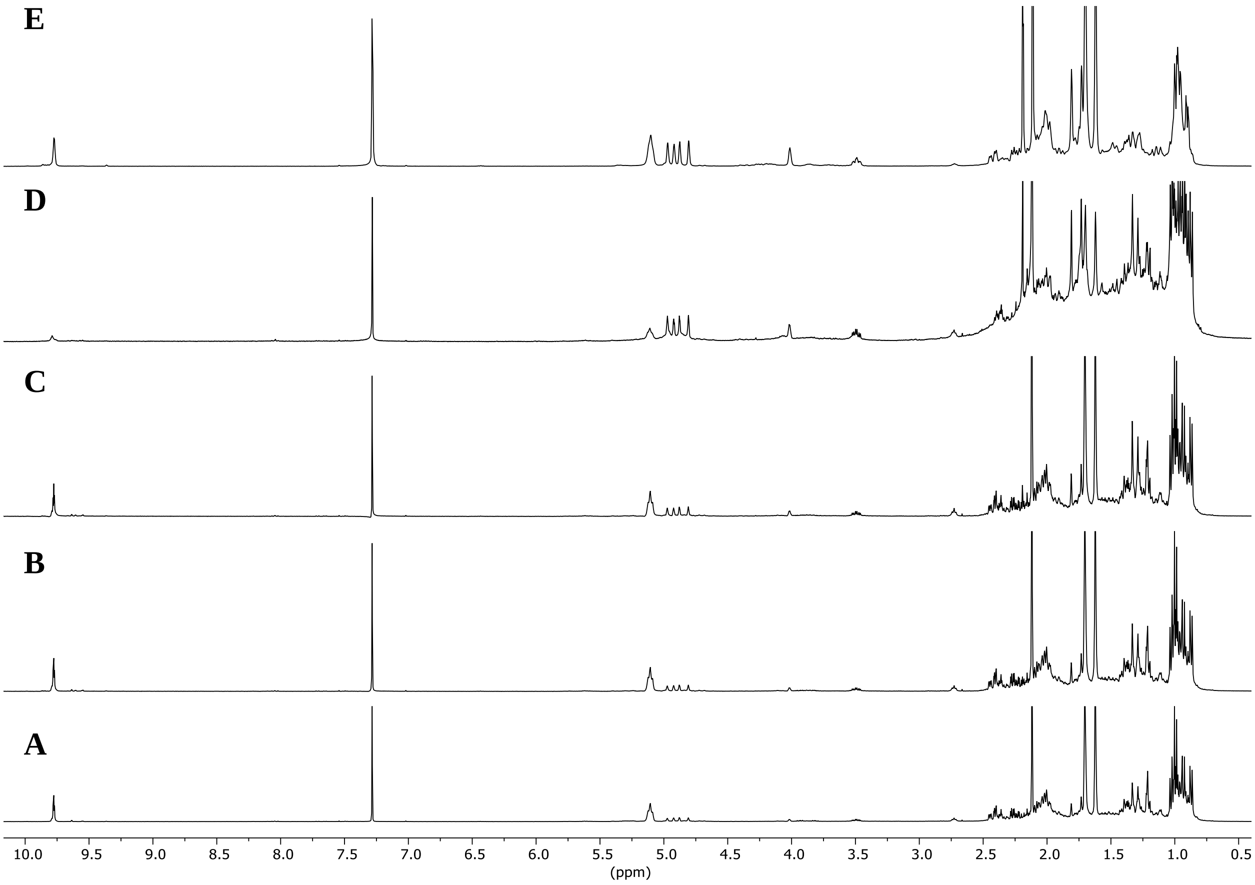

Figure S4: ^{1}H NMR spectra in water-saturated $CDCl_3$ of a solution of citronellal (75 mM), acetic acid (29 mM) recorded after preparation (A), after 1.5 h (B), 3 h (C), 24 h (D), 72 h (E) at 60 °C.

Citronellal isomerization: experiment with capsule [$\mathbf{1}_6$.$(H_2O)_8$]

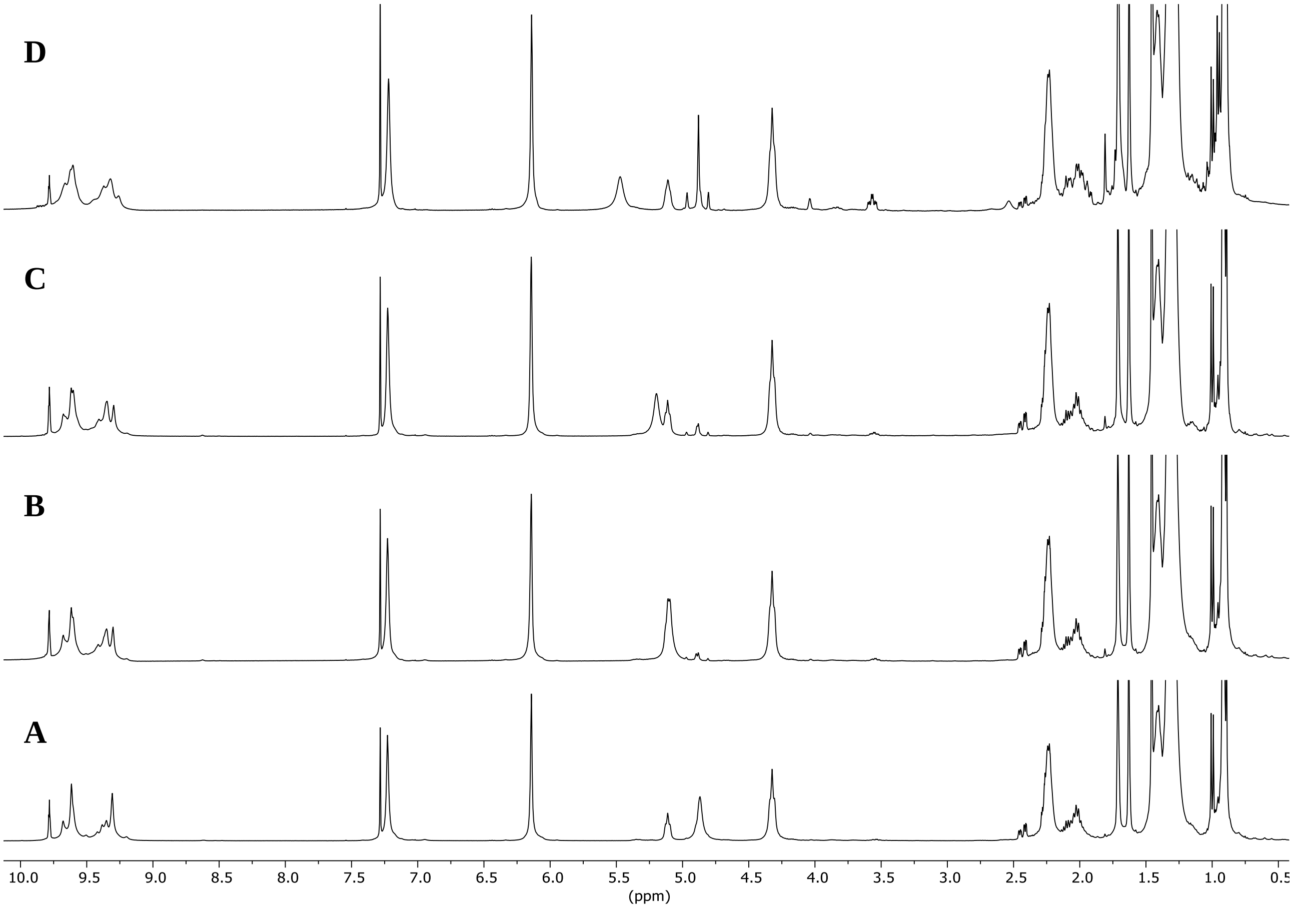

Figure S5: ^{1}H NMR spectra in water-saturated $CDCl_3$ of a solution of citronellal (75 mM), [$\mathbf{1}_6$·$(H_2O)_8$] (7.5 mM), recorded after preparation (A), after 1.5 h (B), 3 h (C), 24 h (D) at 60 °C.

<u>Citronellal isomerization:</u> <u>overview after 24 h</u>

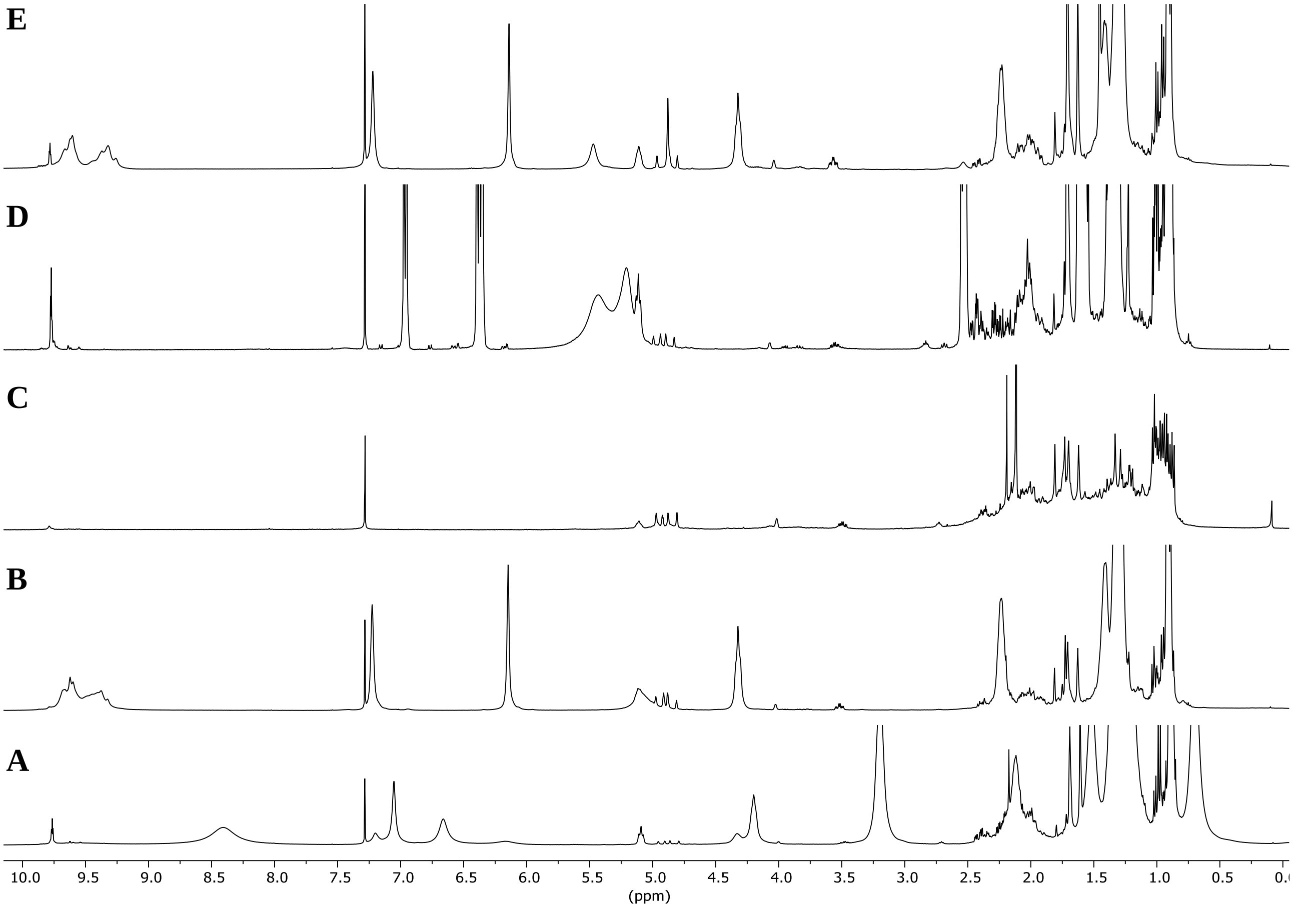

Figure S6: ^{1}H NMR spectra in water-saturated $CDCl_3$. A: citronellal (75 mM), [**1**$_6$·(H_2O)$_8$] (7.5 mM), Bu_4NBr **3** (78 mM); B: citronellal (75 mM), [**1**$_6$·(H_2O)$_8$] (7.5 mM); C: citronellal (75 mM), acetic acid (29 mM); D: citronellal (75 mM), *n*-hexylresorcinol **2** (30 mM); E: citronellal (75 mM), [**1**$_6$·(H_2O)$_8$] (7.5 mM). Spectra recorded after 24 h at 60 °C.

<u>Dehydration of 1,1-diphenylethanol:</u> <u>experiment with capsule [**1**$_6$]</u>

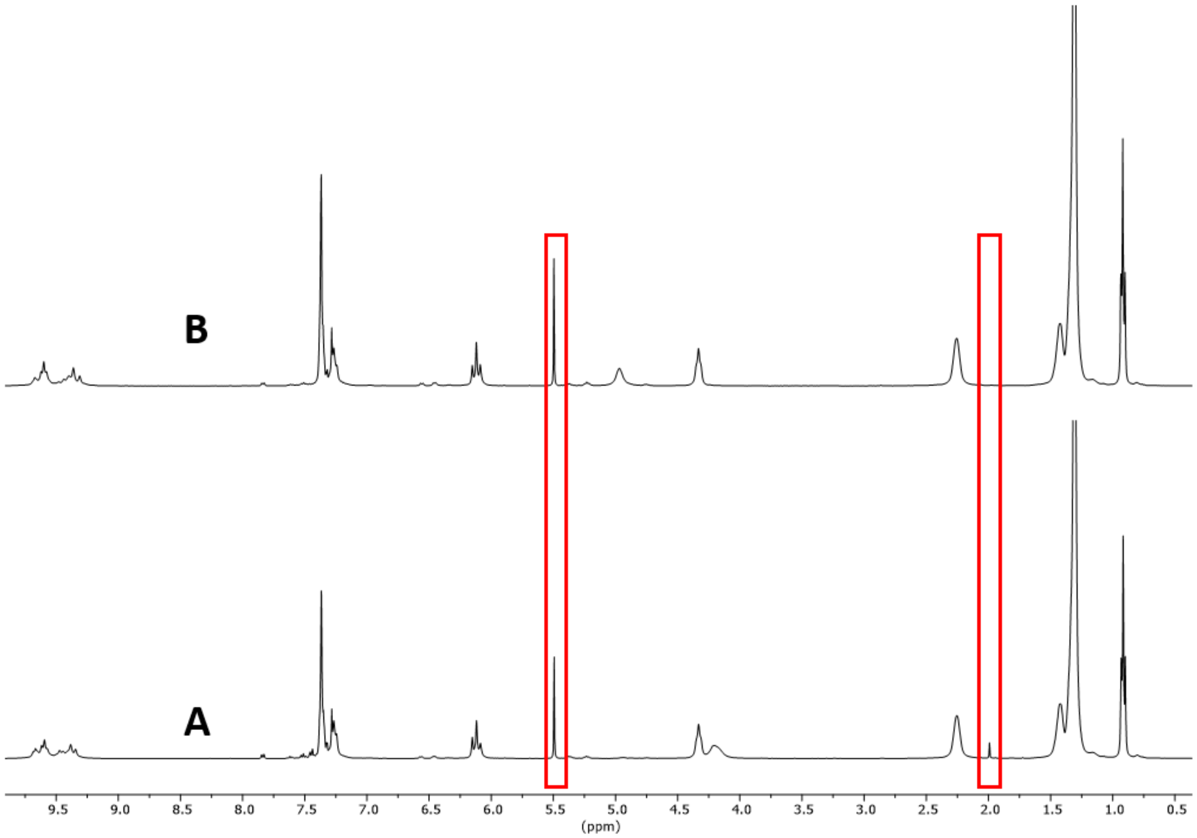

Figure S7: ^{1}H NMR spectra in water-saturated $CDCl_3$ of a solution of 1,1-diphenylethanol (75 mM), [**1**$_6$] (7.5 mM) recorded after 3 h (A) and 20 h (B) at 60 °C.

Dehydration of 1,1-diphenylethanol: control experiments with capsule [$\mathbf{1}_6$] and competitive ammonium guests **3**

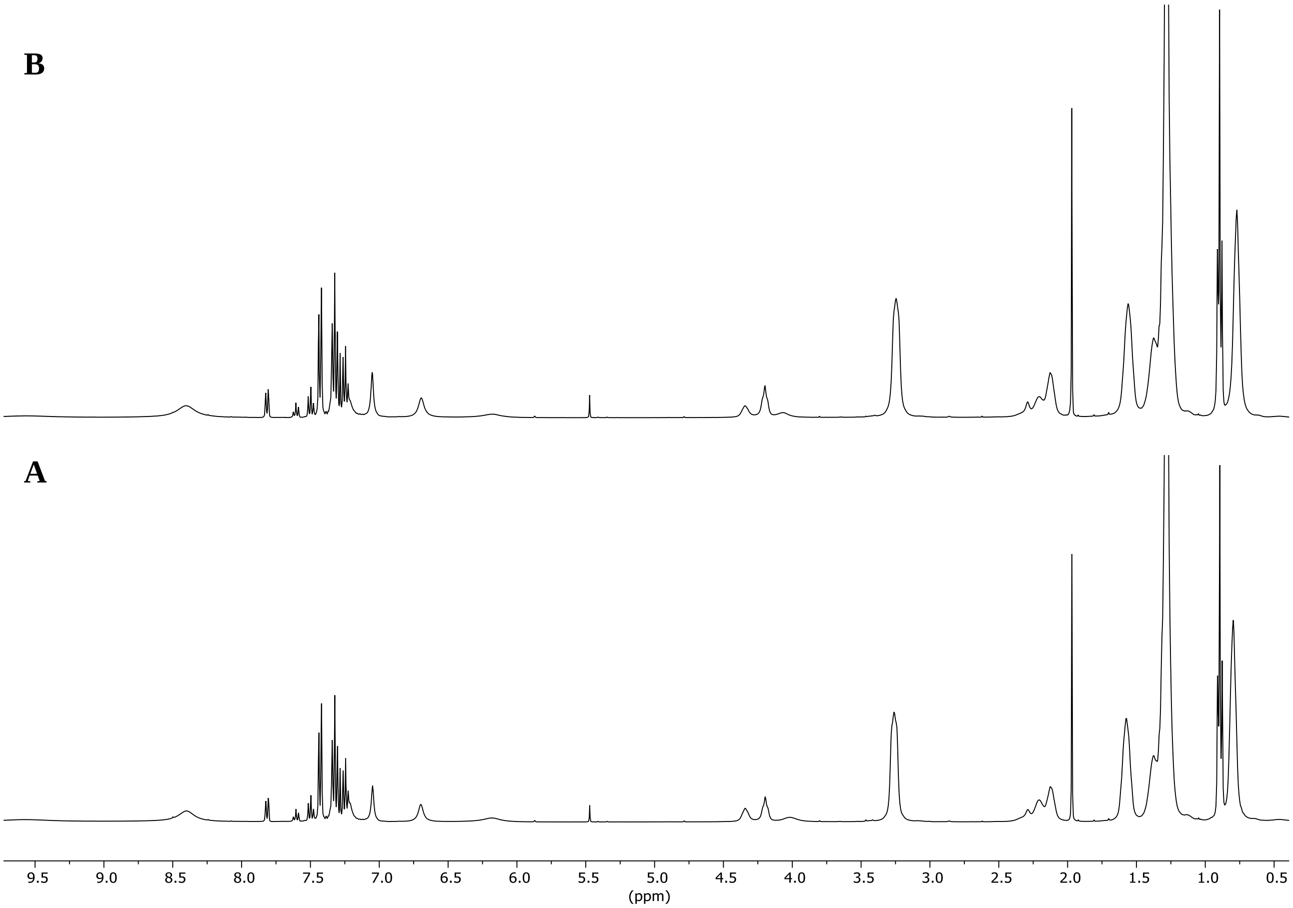

Figure S8: ^{1}H NMR spectra in water-saturated $CDCl_3$ of a solution of 1,1-diphenylethanol (75 mM), [$\mathbf{1}_6$] (7.5 mM), Bu_4NBr **3** (78 mM) recorded after 3 h (A) and 20 h (B) at 60 °C.

<u>Dehydration of 1,1-diphenylethanol:</u> <u>control experiments with 4-*n*-hexylresorcinol (**2**)</u>

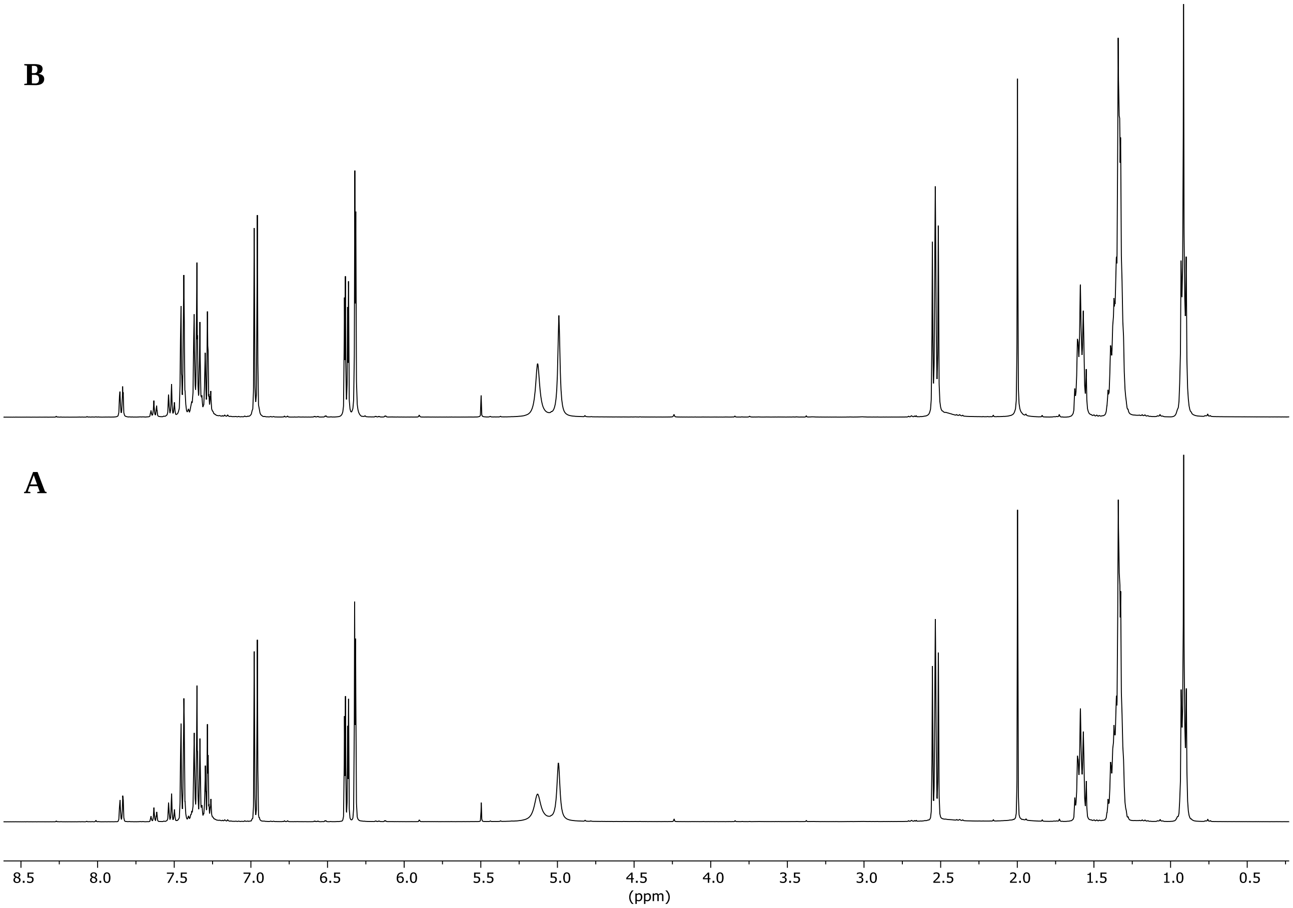

Figure S9: ^{1}H NMR spectra in water-saturated $CDCl_3$ of a solution of 1,1-diphenylethanol (75 mM), n-hexylresorcinol **2** (30 mM) recorded after 3 h (A) and 20 h (B) at 60 °C.

Dehydration of 1,1-diphenylethanol: control experiments with acetic acid

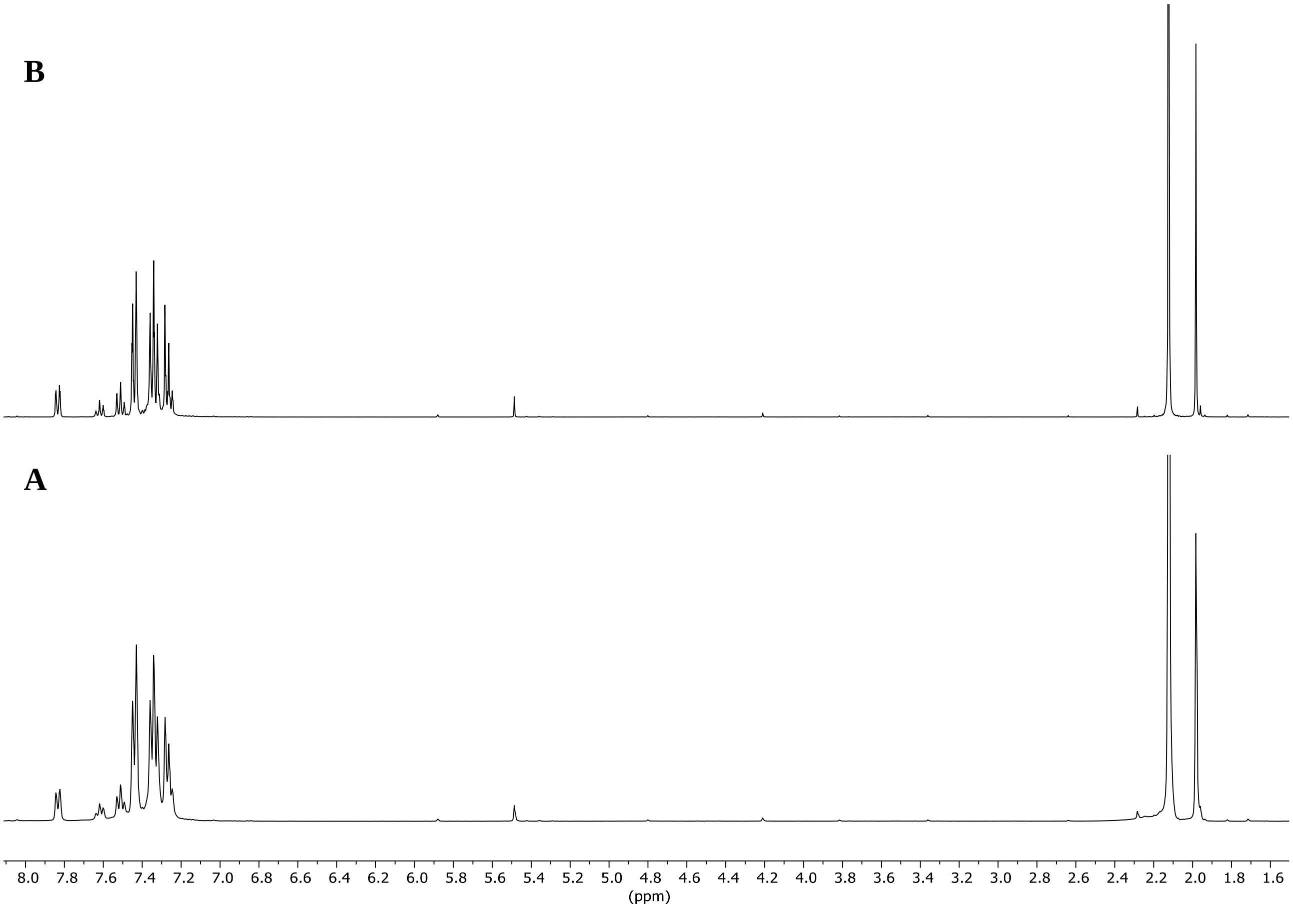

Figure S10: ^{1}H NMR spectra in water-saturated $CDCl_3$ of a solution of 1,1-diphenylethanol (75 mM), acetic acid (29 mM) recorded after 3 h (A) and 20 h (B) at 60 °C.

Dehydration of 1,1-diphenylethanol: experiment with [$\mathbf{1}_6$·(H_2O)$_8$]

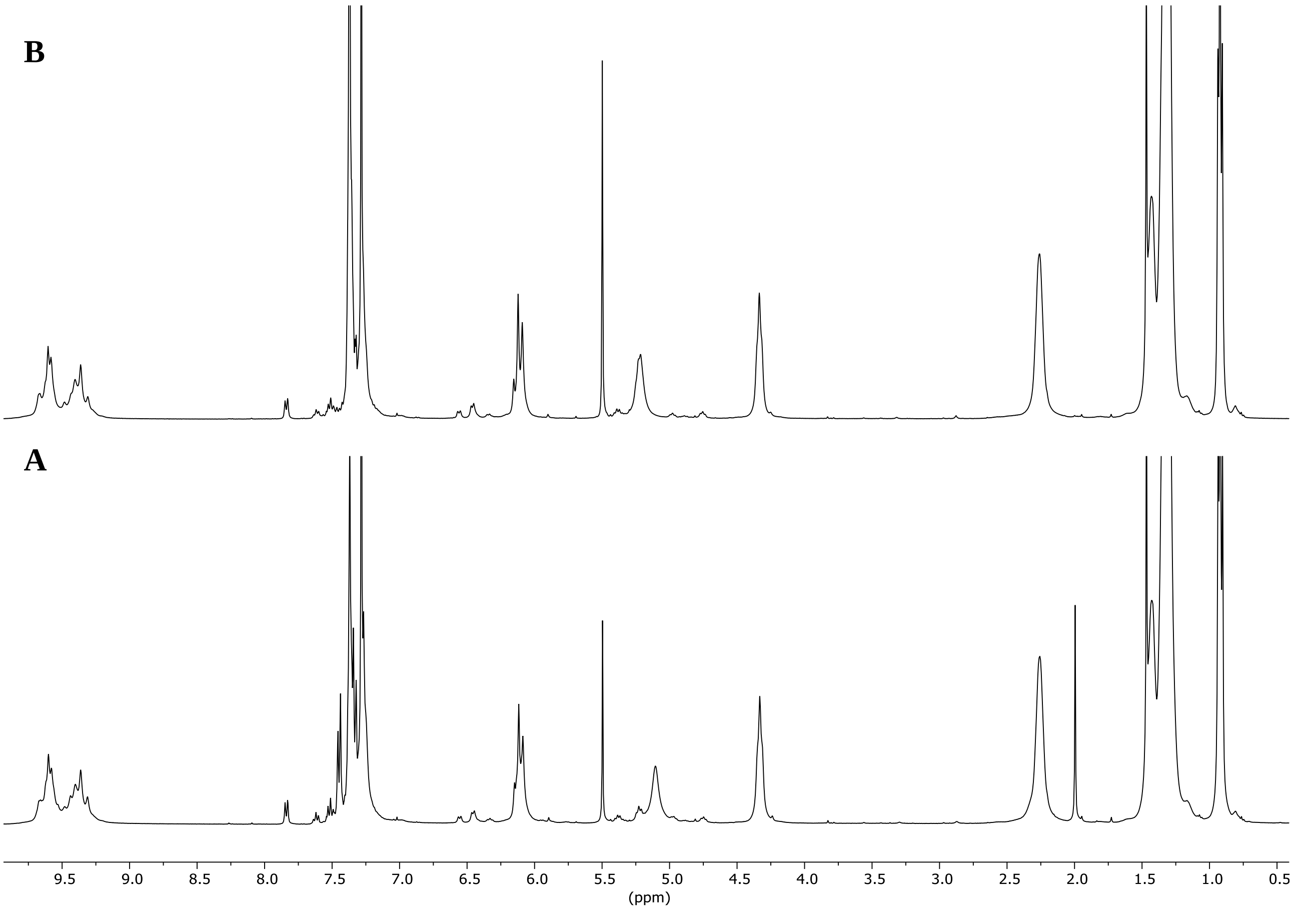

Figure S11: ^{1}H NMR spectra in water-saturated $CDCl_3$ of a solution of 1,1-diphenylethanol (75 mM), [$\mathbf{1}_6$·(H_2O)$_8$] (7.5 mM) recorded after 3 h (A) and 20 h (B) at 60 °C.

Dehydration of 1,1-diphenylethanol: overview after 20 h

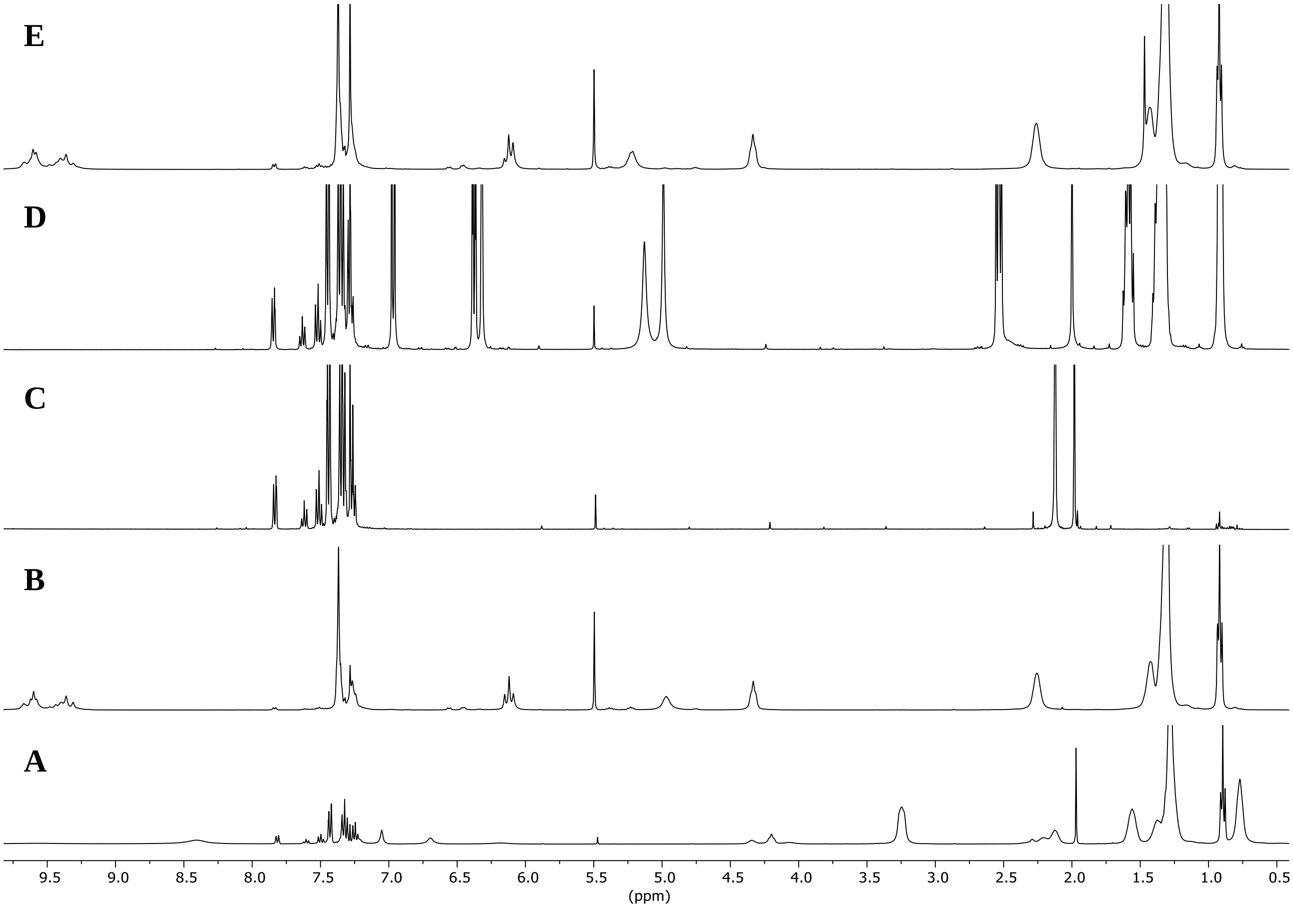

Figure S12:^{1}H NMR spectra in water-saturated $CDCl_3$. A: 1,1-diphenylethanol (75 mM), [**1**$_6$] (7.5 mM), Bu_4NBr **3** (78 mM); B: 1,1-diphenylethanol (75 mM), [**1**$_6$] (7.5 mM); C: 1,1-diphenylethanol (75 mM), acetic acid (29 mM); D: 1,1-diphenylethanol (75 mM), *n*-hexylresorcinol **2** (30 mM); E: 1,1-diphenylethanol (75 mM), [**1**$_6$·$(H_2O)_8$] (7.5 mM). Spectra recorded after 20 h at 60 °C.

α-Pinene isomerization: experiment with capsule [$\mathbf{1}_6$]

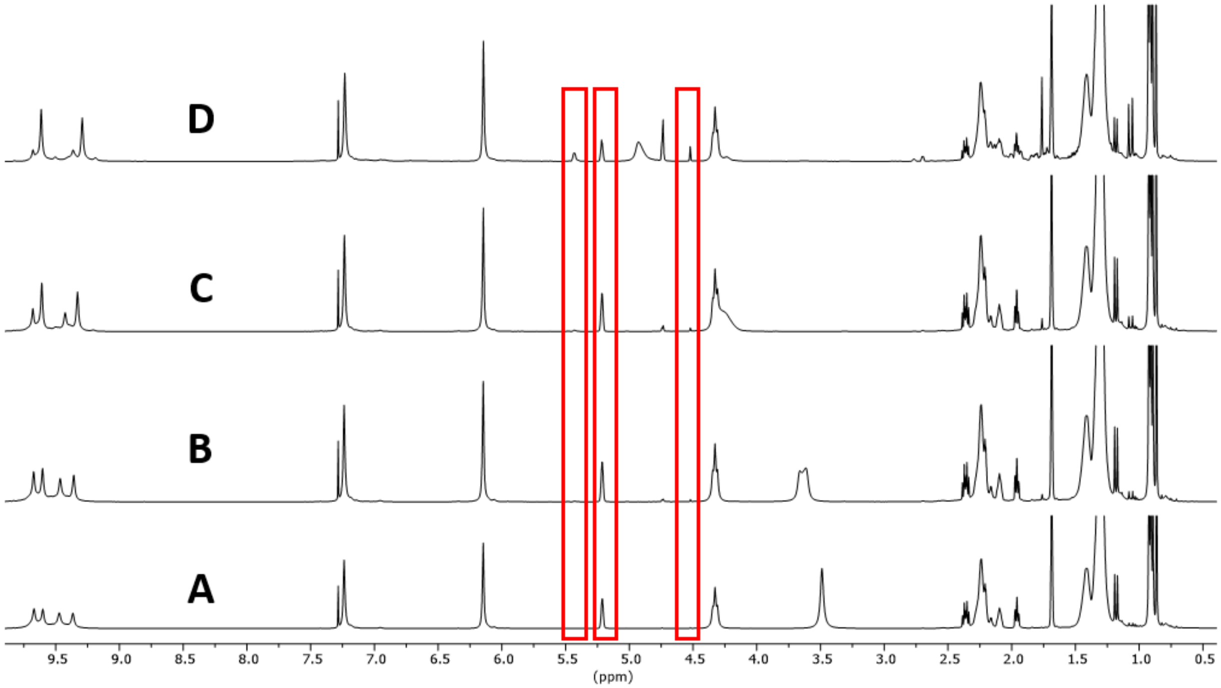

Figure S13: ^{1}H NMR spectra in water-saturated $CDCl_3$ of a solution of α-pinene (75 mM), [$\mathbf{1}_6$] (7.5 mM), recorded after preparation (A), after 1.5 h (B), 3 h (C), 24 h (D) at 60 °C.

α-Pinene isomerization: control experiments with capsule [$\mathbf{1}_6$] and competitive ammonium guests **3**

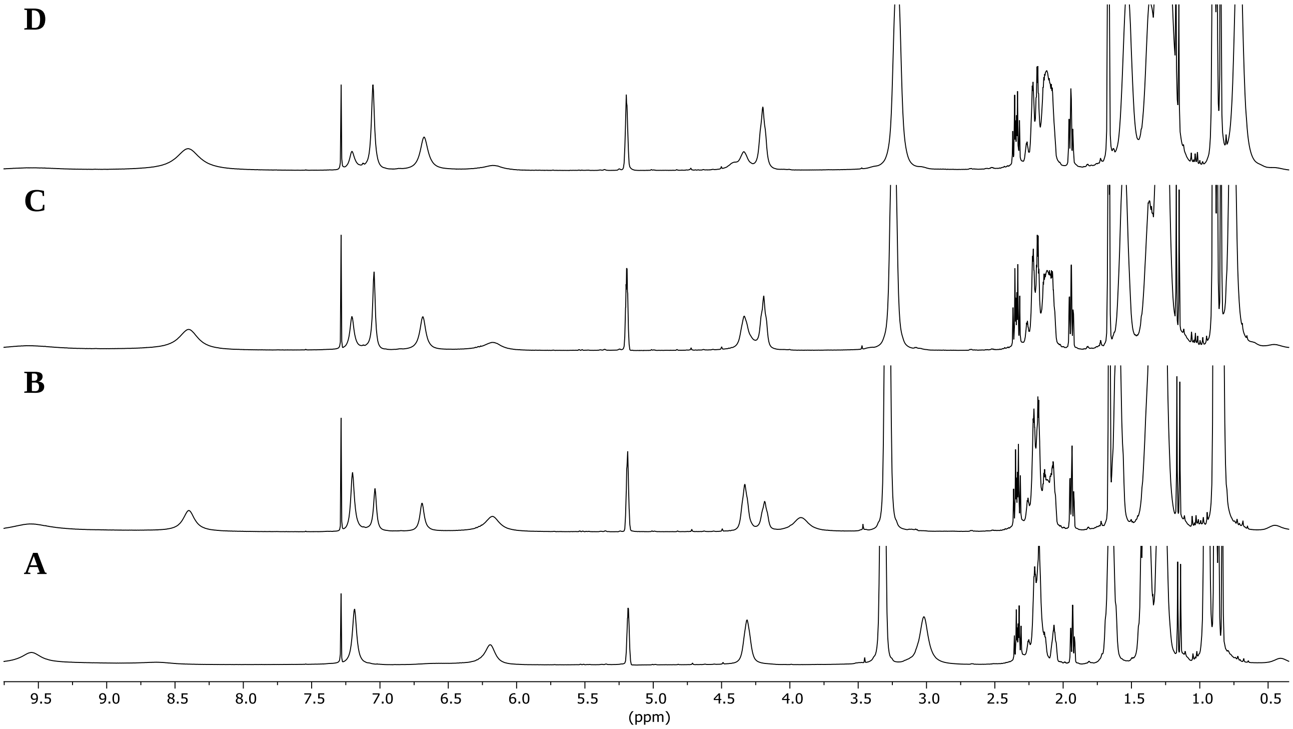

Figure S14: ^{1}H NMR spectra in water-saturated $CDCl_3$ of a solution of α-pinene (75 mM), [$\mathbf{1}_6$] (7.5 mM), Bu_4NBr **3** (78 mM) recorded after preparation (A), after 1.5 h (B), 3 h (C), 24 h (D) at 60 °C.

α-Pinene isomerization: control experiments with 4-*n*-hexyl-resorcinol **2**

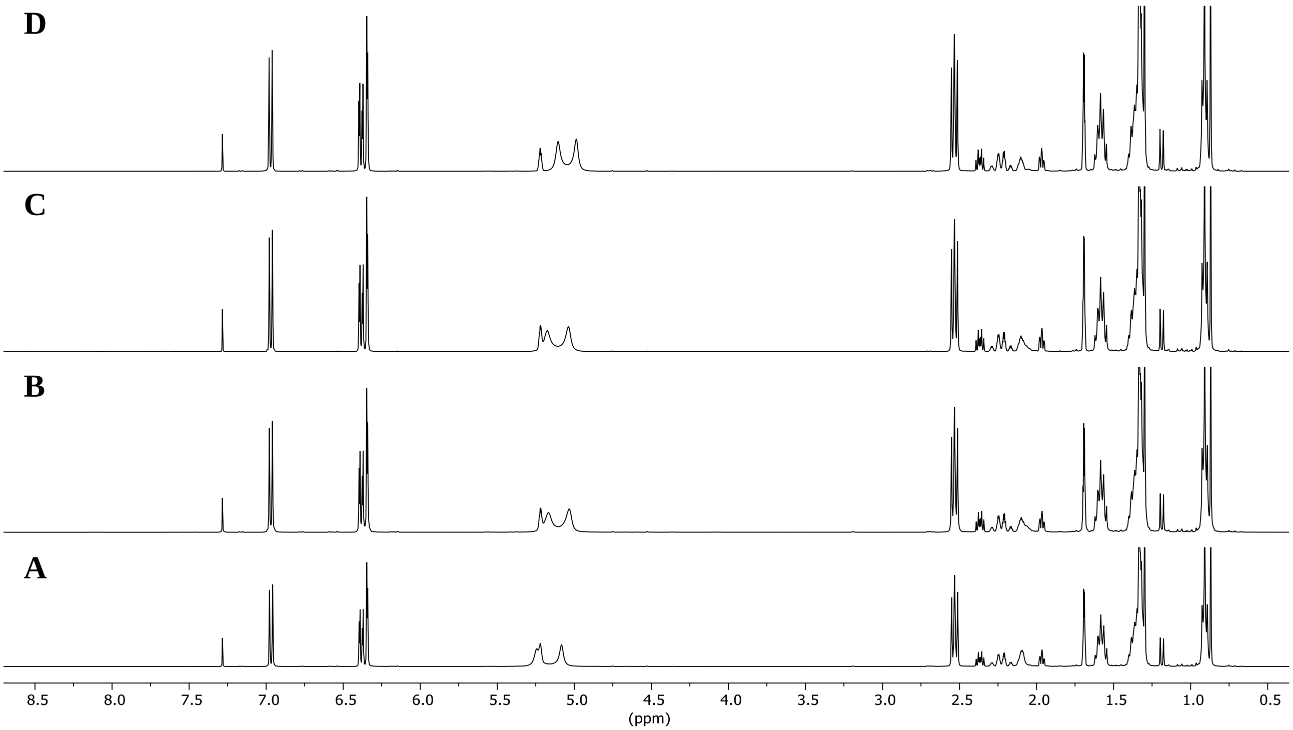

Figure S15: ^{1}H NMR spectra in water-saturated $CDCl_3$ of a solution of α-pinene (75 mM), n-hexylresorcinol **2** (30 mM), recorded after preparation (A), after 1.5 h (B), 3 h (C), 24 h (D) at 60 °C.

α-Pinene isomerization: control experiments with acetic acid

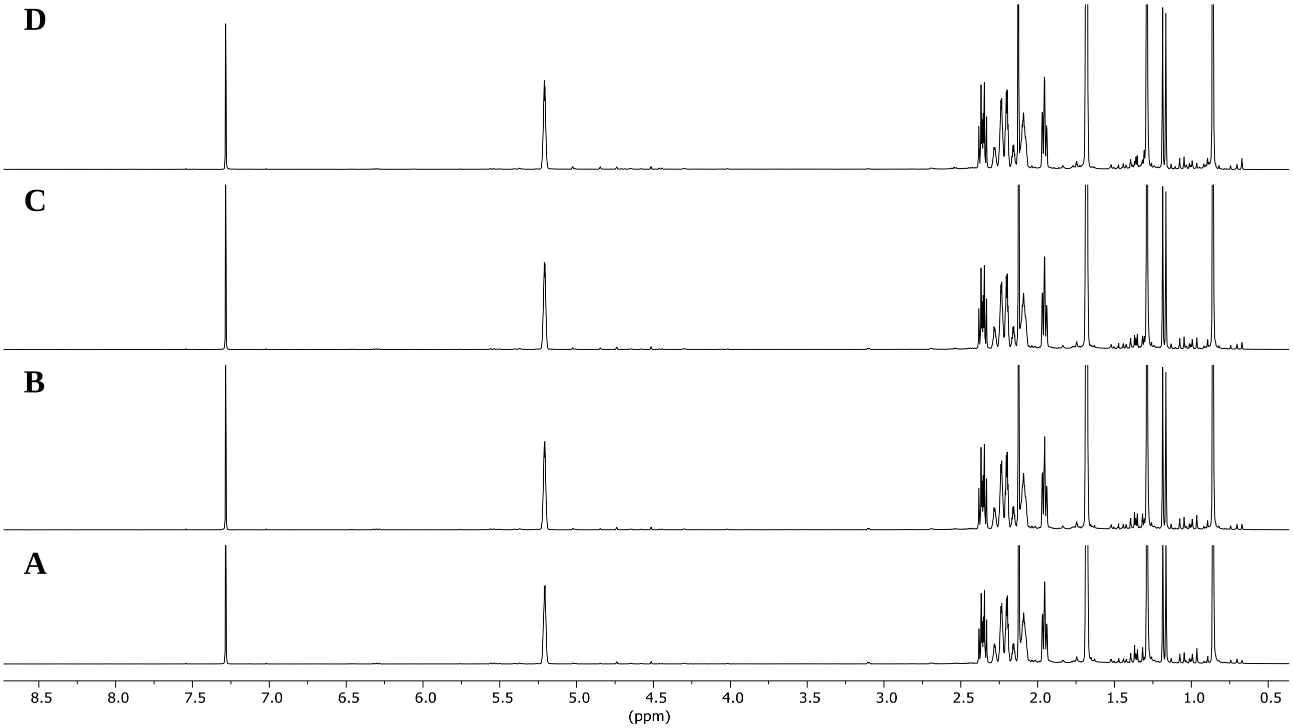

Figure S16:^{1}H NMR spectra in water-saturated $CDCl_3$ of a solution of α-pinene (75 mM), acetic acid (29 mM), recorded after preparation (A), after 1.5 h (B), 3 h (C), 24 h (D) at 60 °C.

α-Pinene isomerization: experiment with capsule [$\mathbf{1}_6$·(H_2O)$_8$]

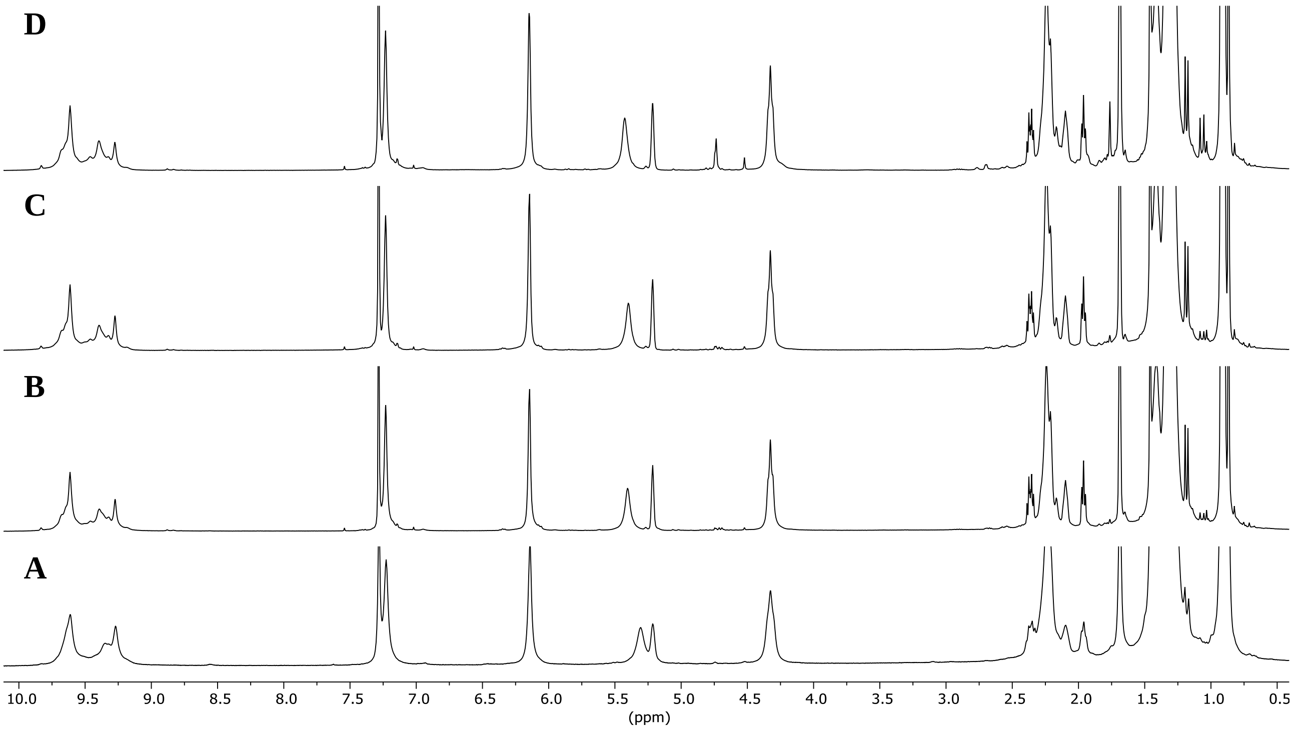

Figure S17: ^{1}H NMR spectra in water-saturated $CDCl_3$ of a solution of α-pinene (75 mM), [$\mathbf{1}_6$·(H_2O)$_8$] (7.5 mM), recorded after preparation (A), after 1.5 h (B), 3 h (C), 24 h (D) at 60 °C.

α-Pinene isomerization: overview after 24 h

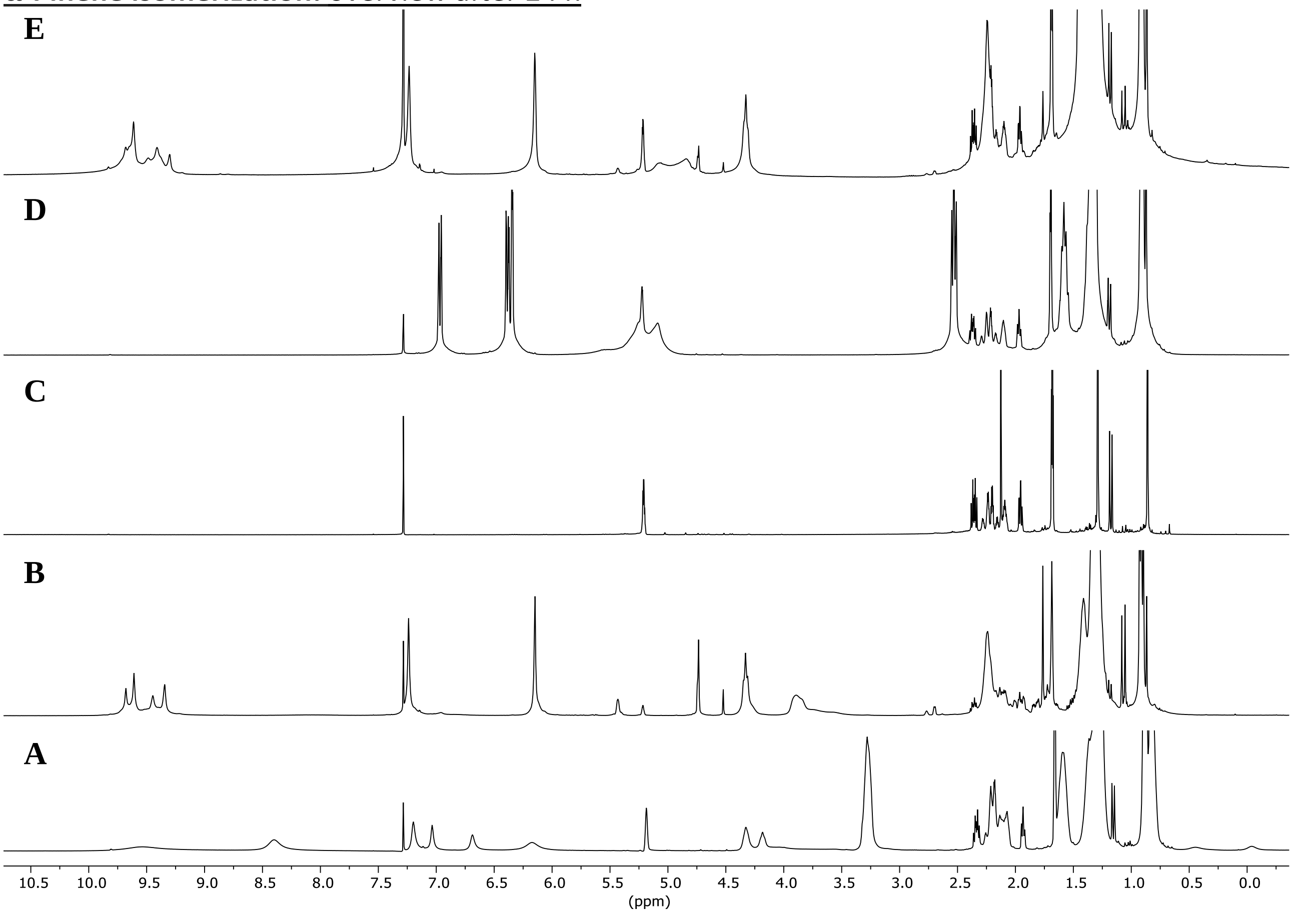

Figure S18: ^{1}H NMR spectra in water-saturated $CDCl_3$. A: α-pinene (75 mM), [$\mathbf{1}_6$·$(H_2O)_8$] (7.5 mM), Bu_4NBr **3** (78 mM); B: α-pinene (75 mM), [$\mathbf{1}_6$·$(H_2O)_8$] (7.5 mM); C: α-pinene (75 mM), acetic acid (29 mM); D: α-pinene (75 mM), *n*-hexylresorcinol **2** (30 mM); E: α-pinene (75 mM), [$\mathbf{1}_6$·$(H_2O)_8$] (7.5 mM). Spectra recorded after 24 h at 60 °C.

β-Pinene isomerization: Experiment with capsule [$\mathbf{1}_6$]

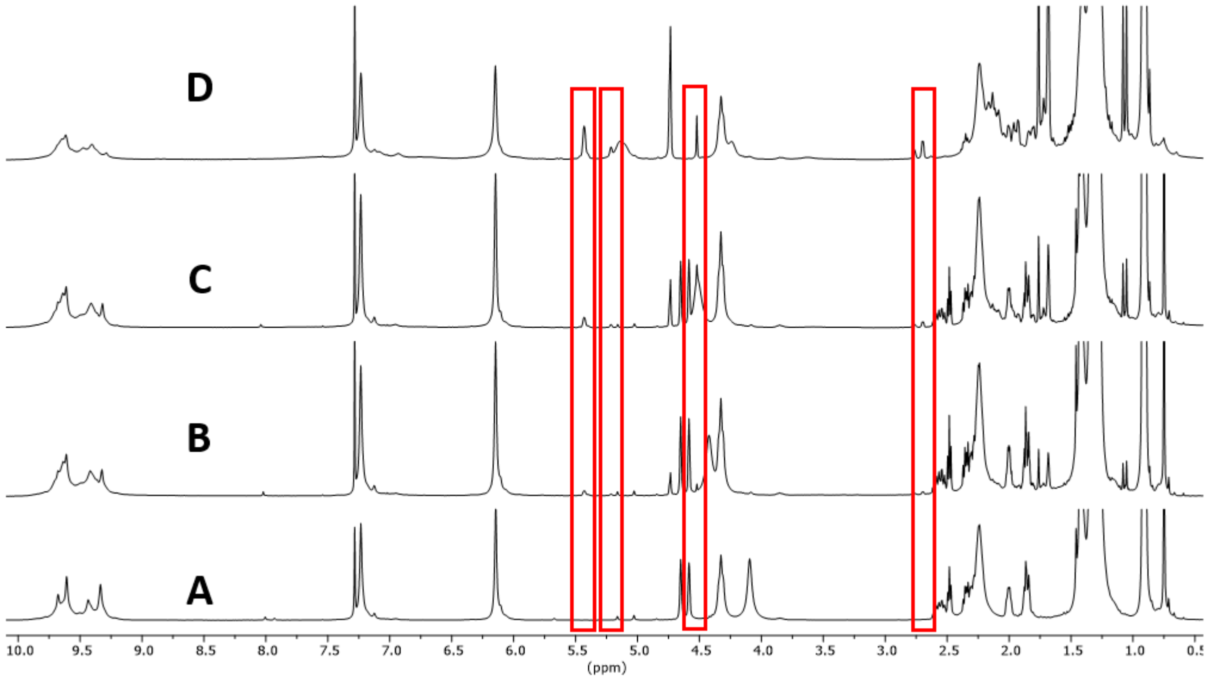

Figure S19: ^{1}H NMR spectra in water-saturated $CDCl_3$ of a solution of β-pinene (75 mM), [$\mathbf{1}_6$] (7.5 mM), recorded after preparation (A), after 1.5 h (B), 3 h (C), 24 h (D) at 60 °C.

β-Pinene isomerization: control experiments with capsule [**1**$_6$] and competitive ammonium guests **3**

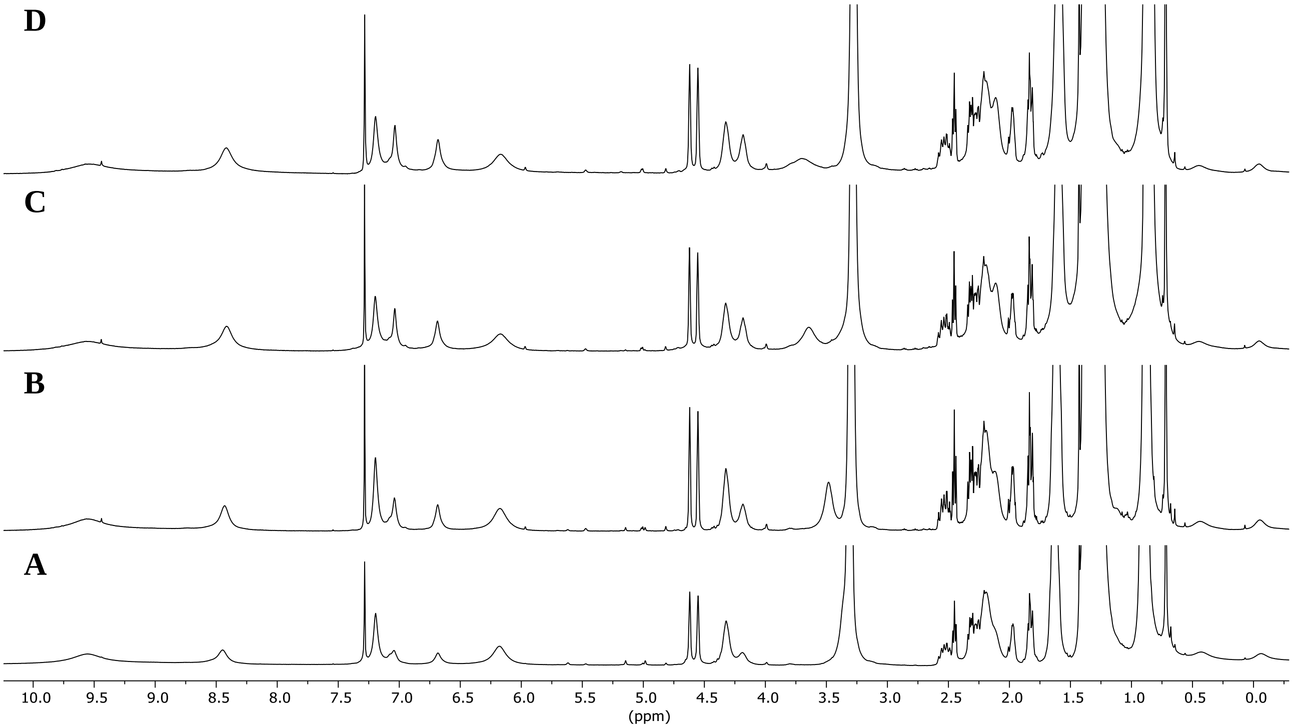

Figure S20:^{1}H NMR spectra in water-saturated $CDCl_3$ of a solution of β-pinene (75 mM), [**1**$_6$] (7.5 mM), Bu_4NBr **3** (78 mM) recorded after preparation (A), after 1.5 h (B), 3 h (C), 24 h (D) at 60 °C.

β-Pinene isomerization: control experiments with 4-*n*-hexyl-resorcinol **2**

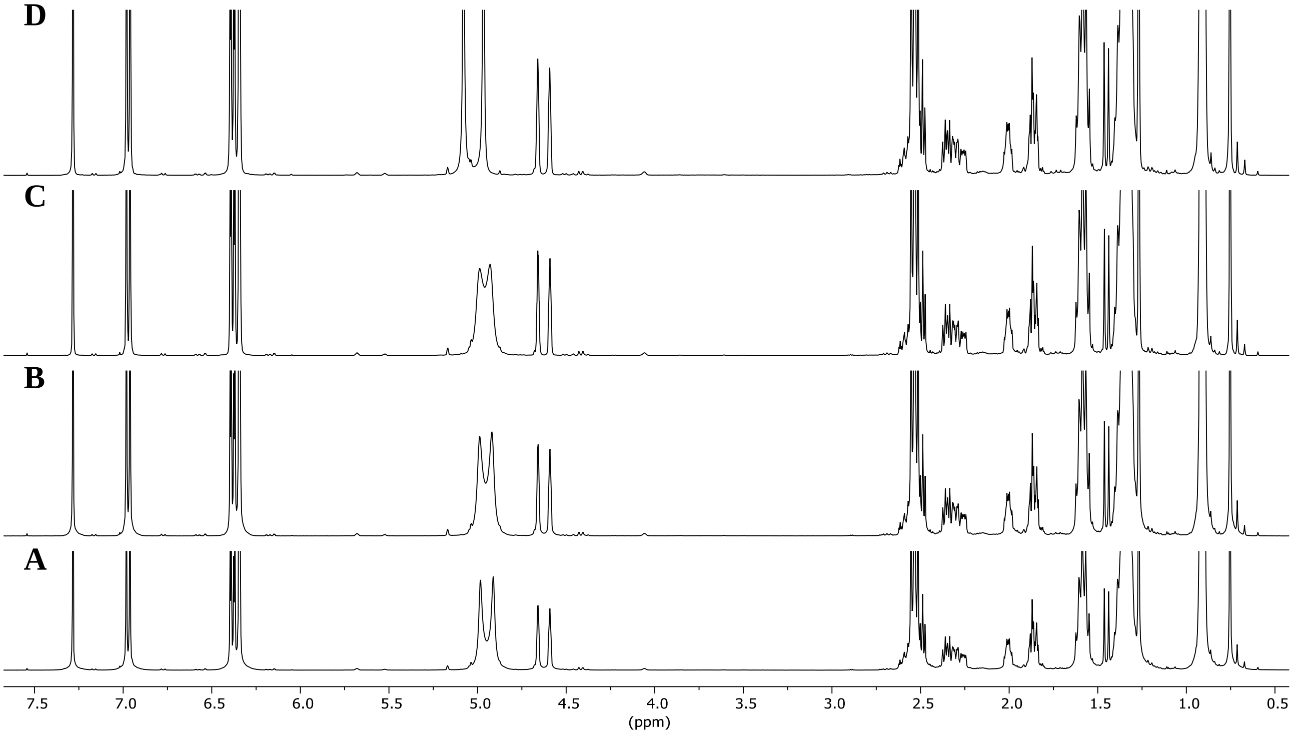

Figure S21:^{1}H NMR spectra in water-saturated $CDCl_3$ of a solution of β-pinene (75 mM), n-hexylresorcinol **2** (30 mM), recorded after preparation (A), after 1.5 h (B), 3 h (C), 24 h (D) at 60 °C.

β-Pinene isomerization: control experiments with acetic acid

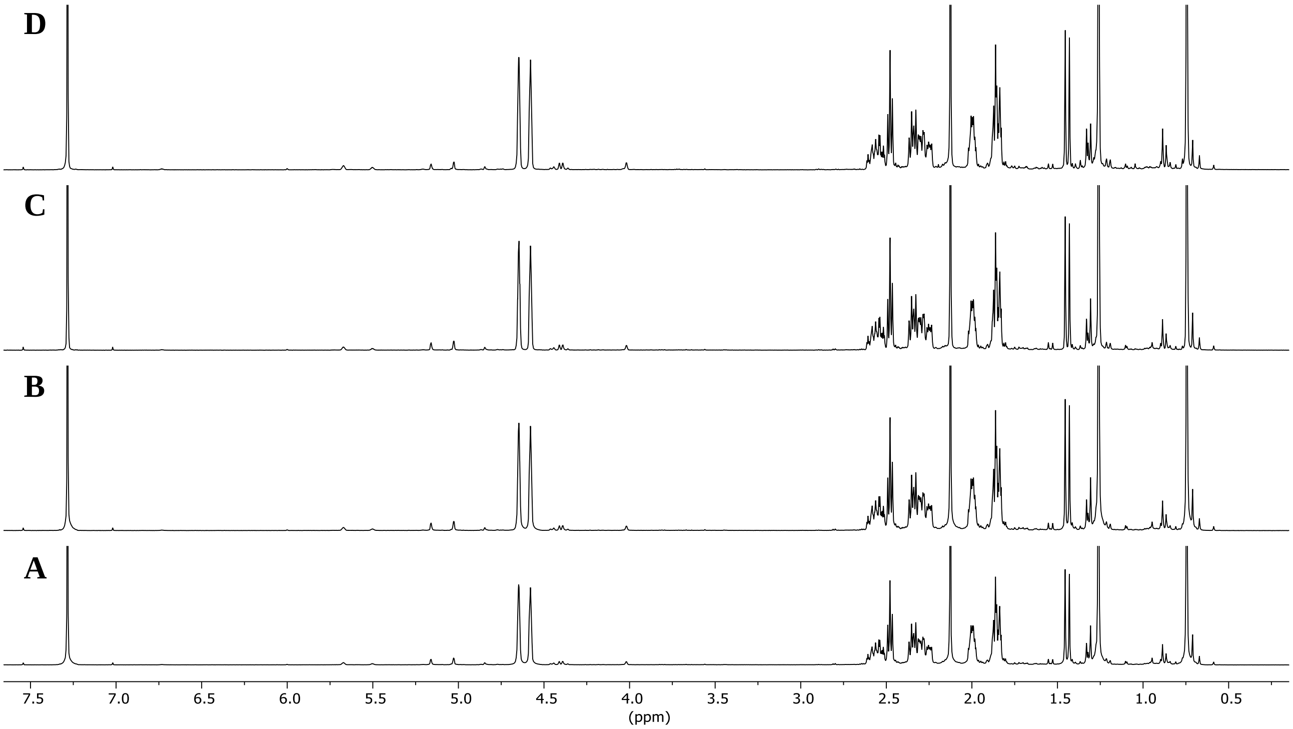

Figure S22: ^{1}H NMR spectra in water-saturated $CDCl_3$ of a solution of β-pinene (75 mM), acetic acid (29 mM), recorded after preparation (A), after 1.5 h (B), 3 h (C), 24 h (D) at 60 °C.

β-Pinene isomerization: experiment with capsule [**1**$_6$·(H$_2$O)$_8$]

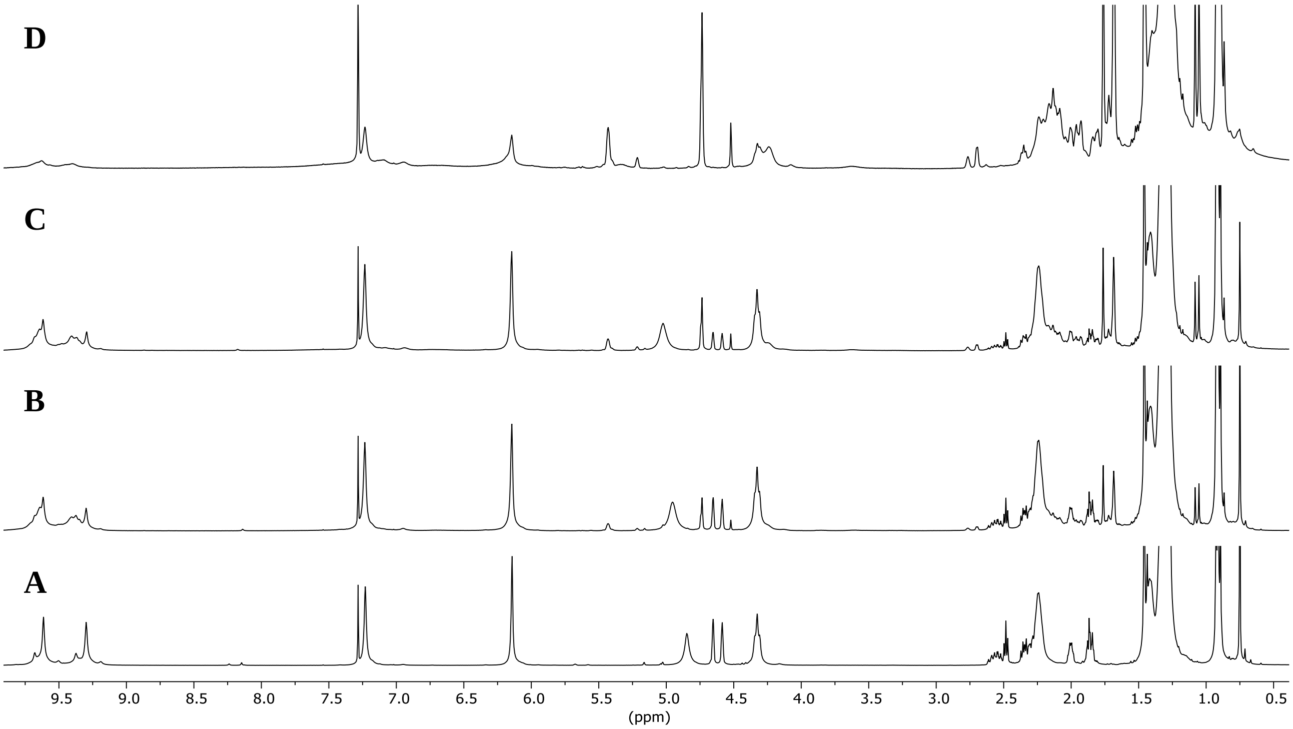

Figure S23: ^{1}H NMR spectra in water-saturated $CDCl_3$ of a solution of β-pinene (75 mM), [**1**$_6$·(H$_2$O)$_8$] (7.5 mM), recorded after preparation (A), after 1.5 h (B), 3 h (C), 24 h (D) at 60 °C.

__**β-Pinene isomerization:** overview after 24 h__

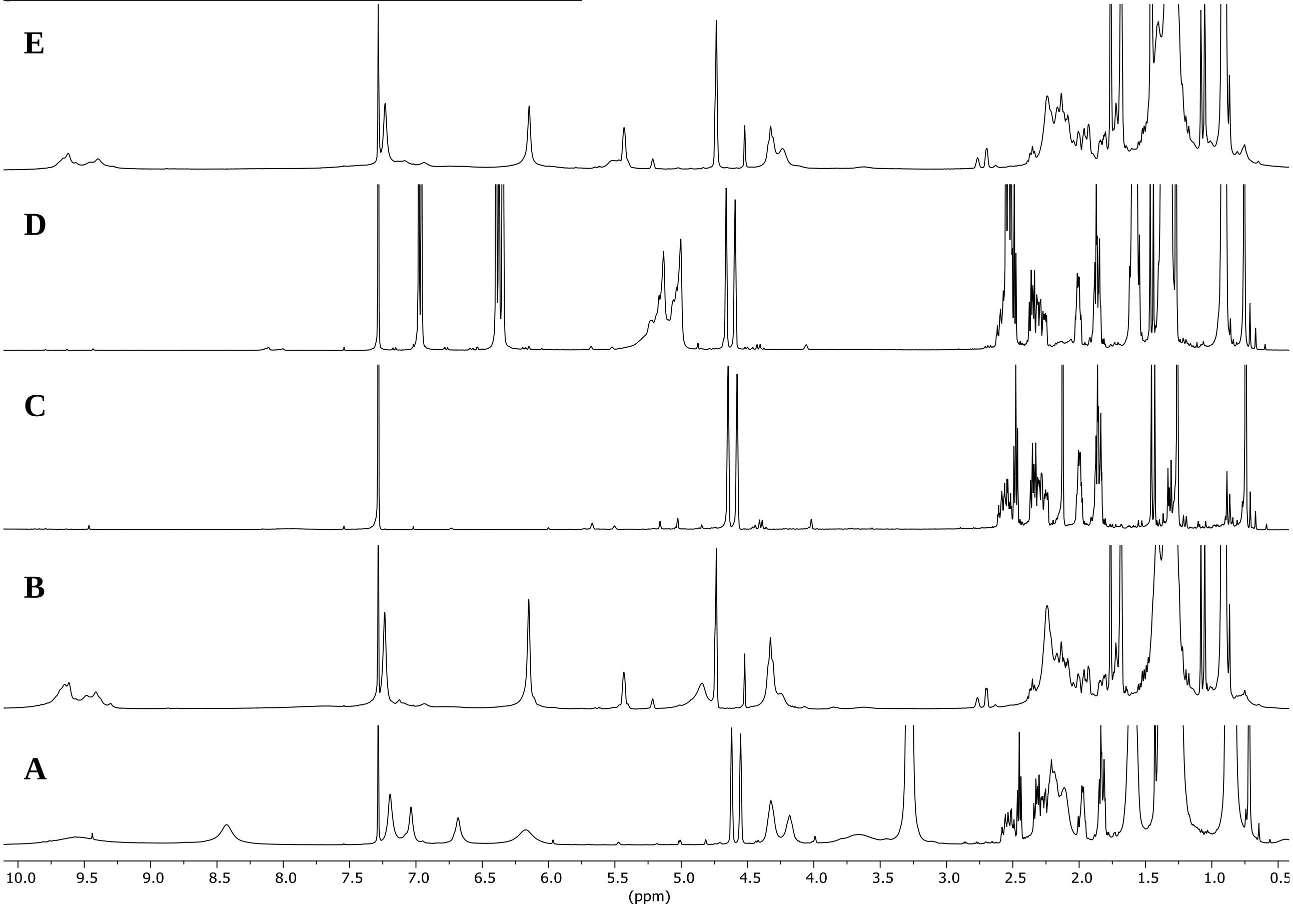

Figure S24:^{1}H NMR spectra in water-saturated $CDCl_3$. A: β-pinene (75 mM), [$\mathbf{1}_6$] (7.5 mM), Bu_4NBr **3** (78 mM); B: β-pinene (75 mM), [$\mathbf{1}_6$] (7.5 mM); C: β-pinene (75 mM), acetic acid (29 mM); D: β-pinene (75 mM), *n*-hexylresorcinol **2** (30 mM); E: β-pinene (75 mM), [$\mathbf{1}_6 \cdot (H_2O)_8$] (7.5 mM). Spectra recorded after 24 h at 60 °C.

β-Pinene isomerization at room temperature: experiment with capsule [**1**$_6$]

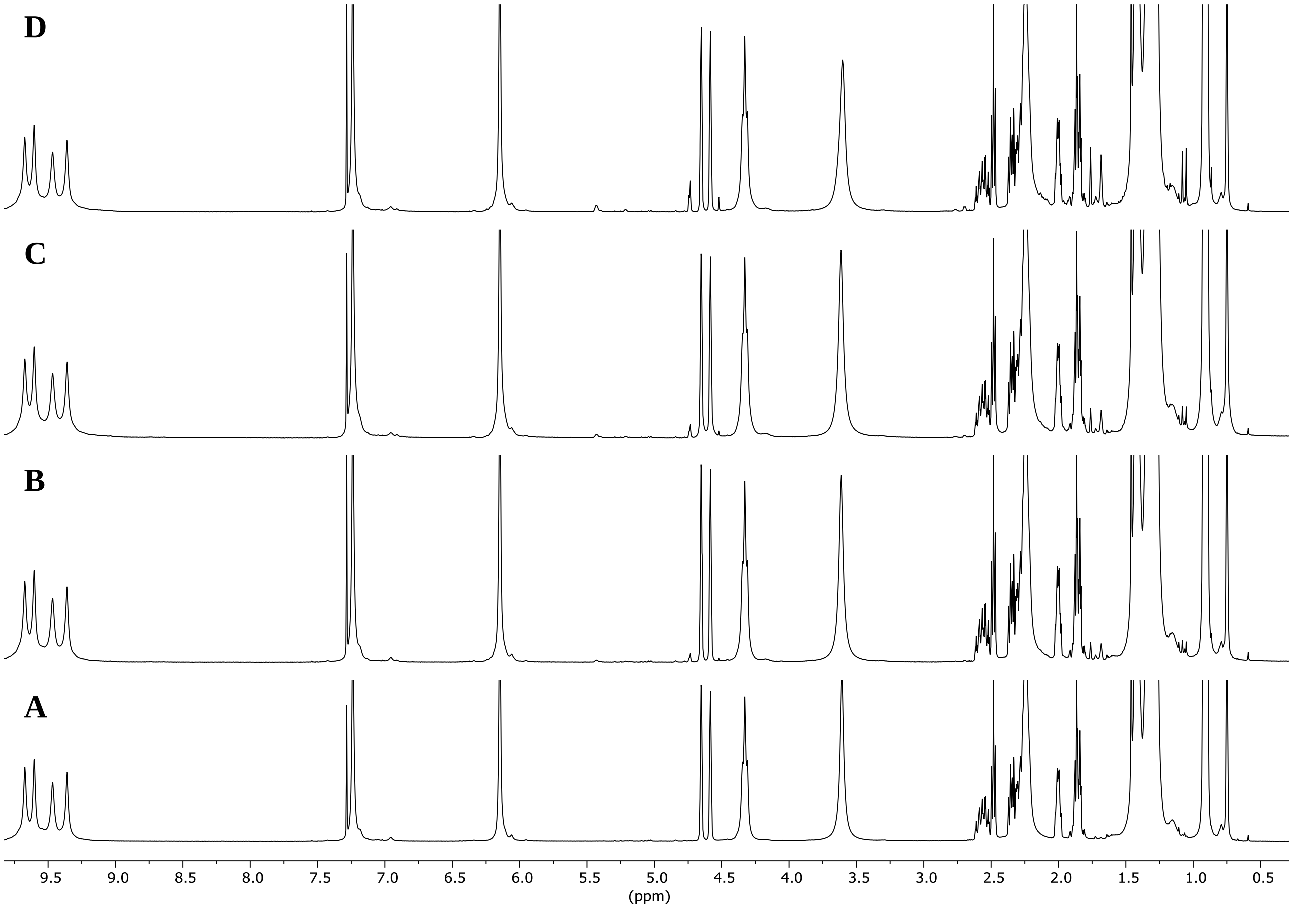

Figure S25: ^{1}H NMR spectra in water-saturated $CDCl_3$ of a solution of β-pinene (75 mM), [**1**$_6$] (7.5 mM) recorded after 1 h (A), 23 h (B), 35 h (C), 64 h (D) at room temperature.

β-Pinene isomerization at room temperature: control experiments with capsule [**1**$_6$] and competitive ammonium guests **3**

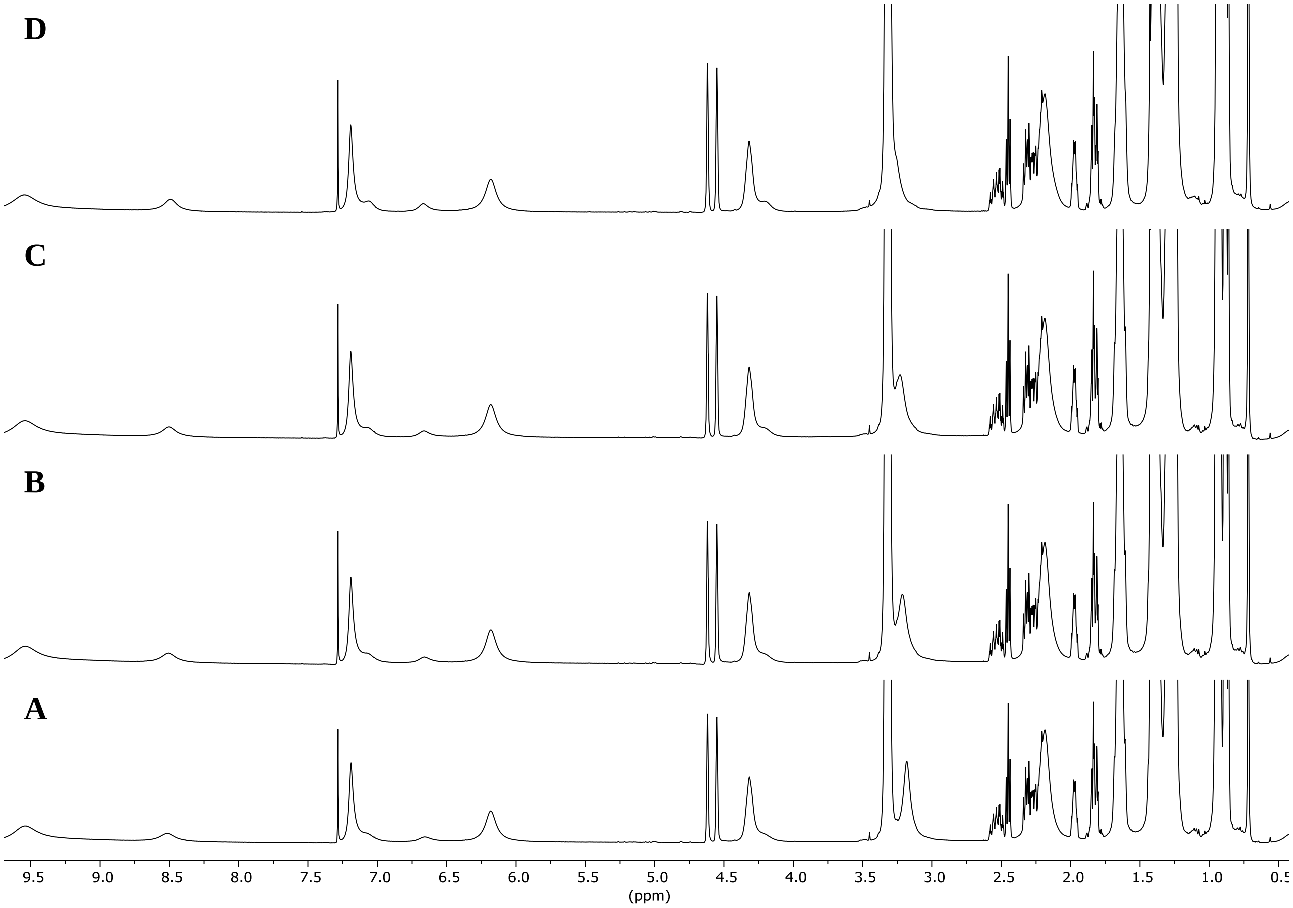

Figure S26:^{1}H NMR spectra in water-saturated $CDCl_3$ of a solution of β-pinene (75 mM), [**1**$_6$] (7.5 mM), Bu_4NBr **3** (78 mM) recorded after 1 h (A), 23 h (B), 35 h (C), 64 h (D) at room temperature.

β-Pinene isomerization at room temperature: experiment with capsule [$\mathbf{1}_6$·(H_2O)$_8$]

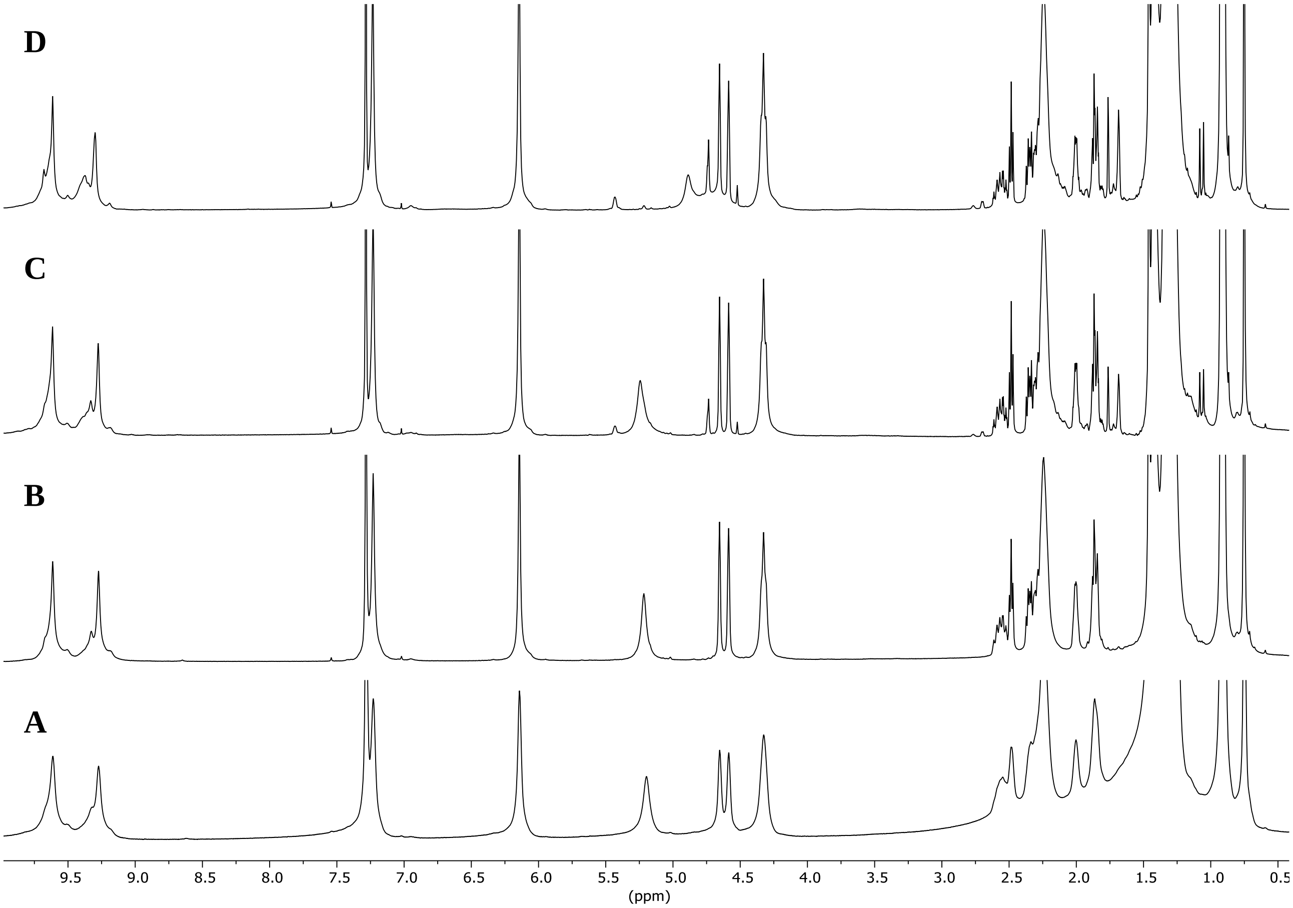

Figure S27:^{1}H NMR spectra in water-saturated $CDCl_3$ of a solution of β-pinene (75 mM), [$\mathbf{1}_6$·(H_2O)$_8$] (7.5 mM) recorded after 1 h (A), 23 h (B), 35 h (C), 64 h (D) room temperature.

β-Pinene isomerization at room temperature: overview after 64 h

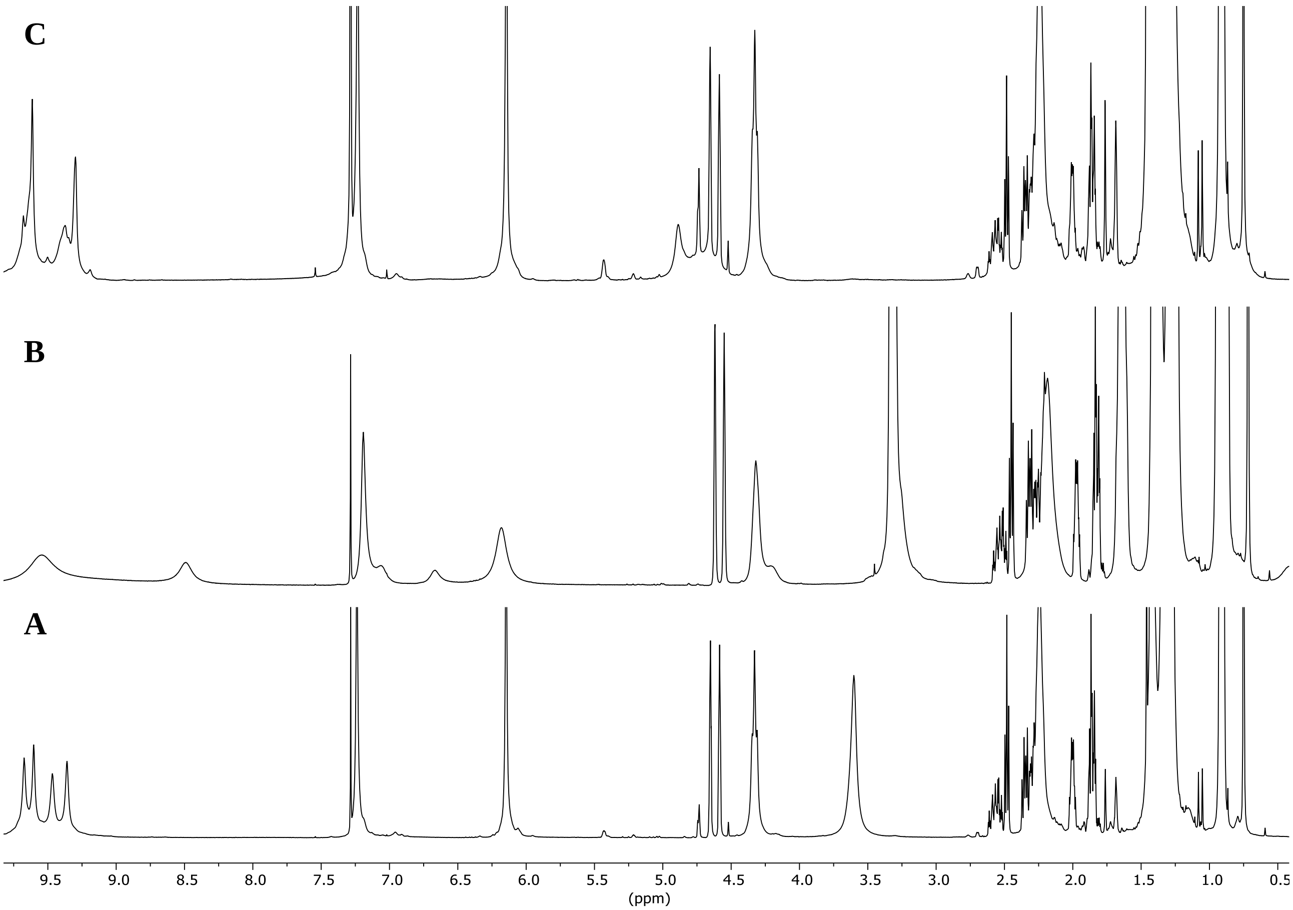

Figure S28: ^{1}H NMR spectra in water-saturated $CDCl_3$. A: β-pinene (75 mM), [**1**$_6^{\cdot}$] (7.5 mM); B: β-pinene (75 mM), [**1**$_6$] (7.5 mM), Bu_4NBr **3** (78 mM); C: β-pinene (75 mM), [**1**$_6^{\cdot}$$(H_2O)_8$] (7.5 mM). Spectra recorded after 64 h at room temperature.

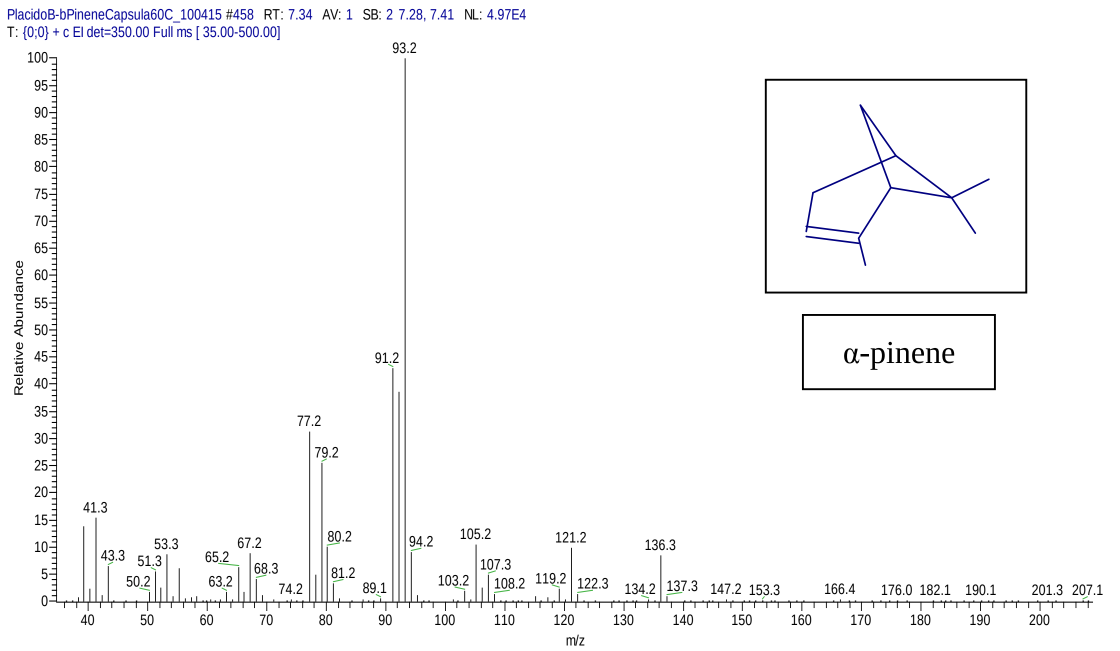

Figure S29: EIMS spectrum of α-pinene.

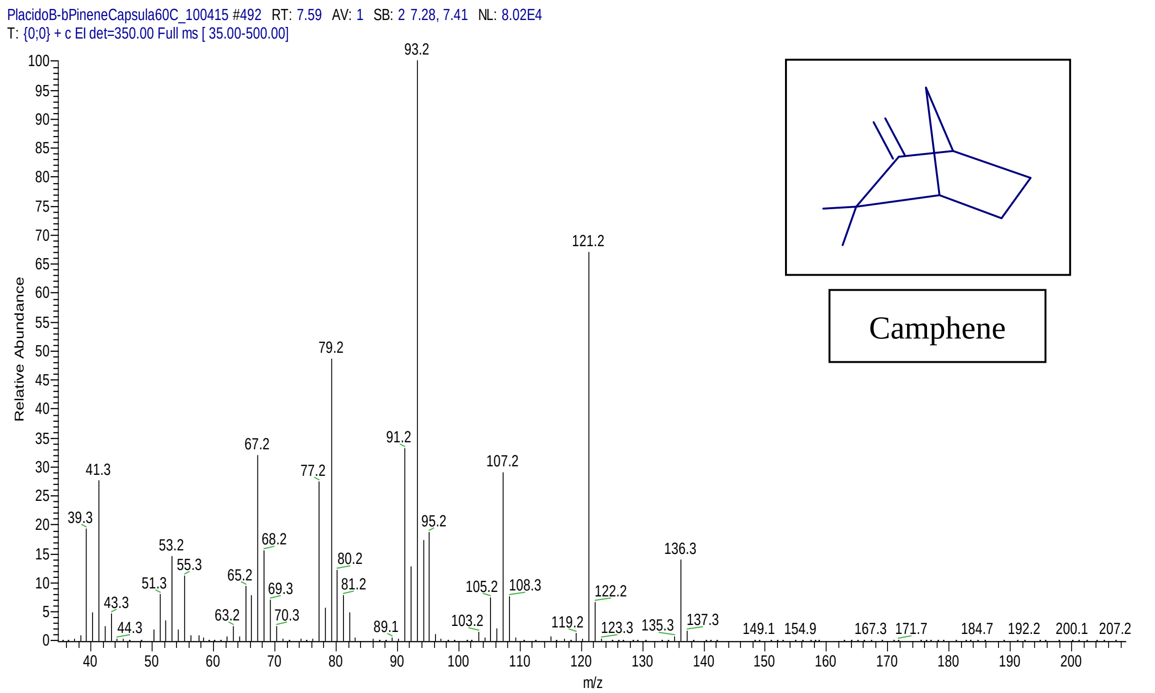

Figure S30: EIMS spectrum of camphene.

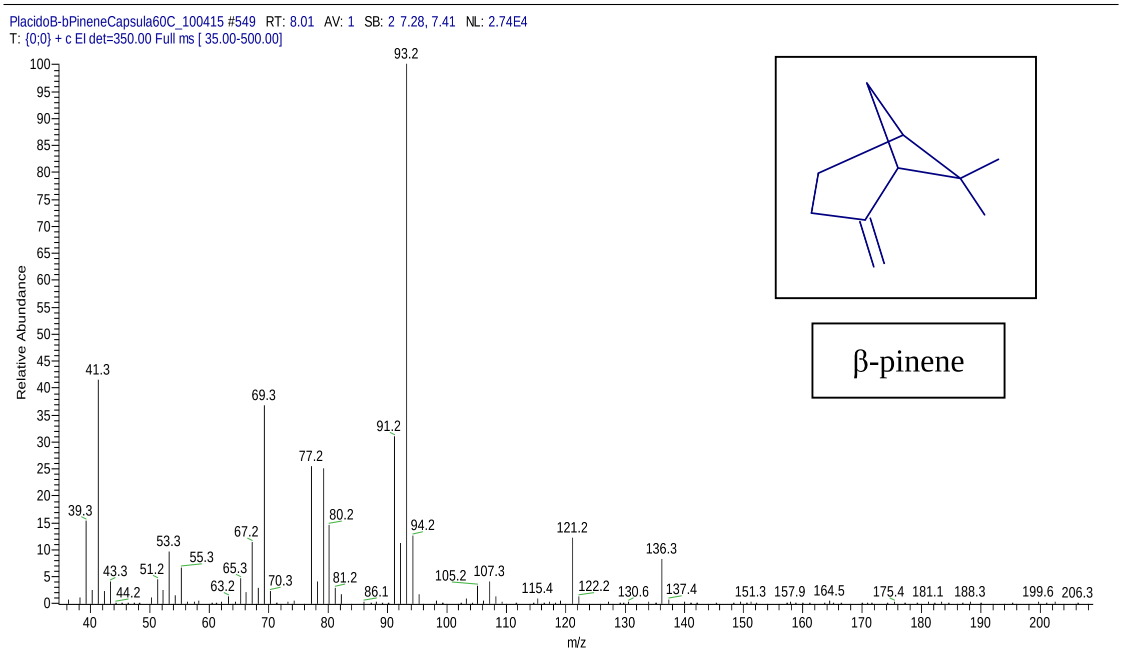

Figure S31: EIMS spectrum of β-pinene.

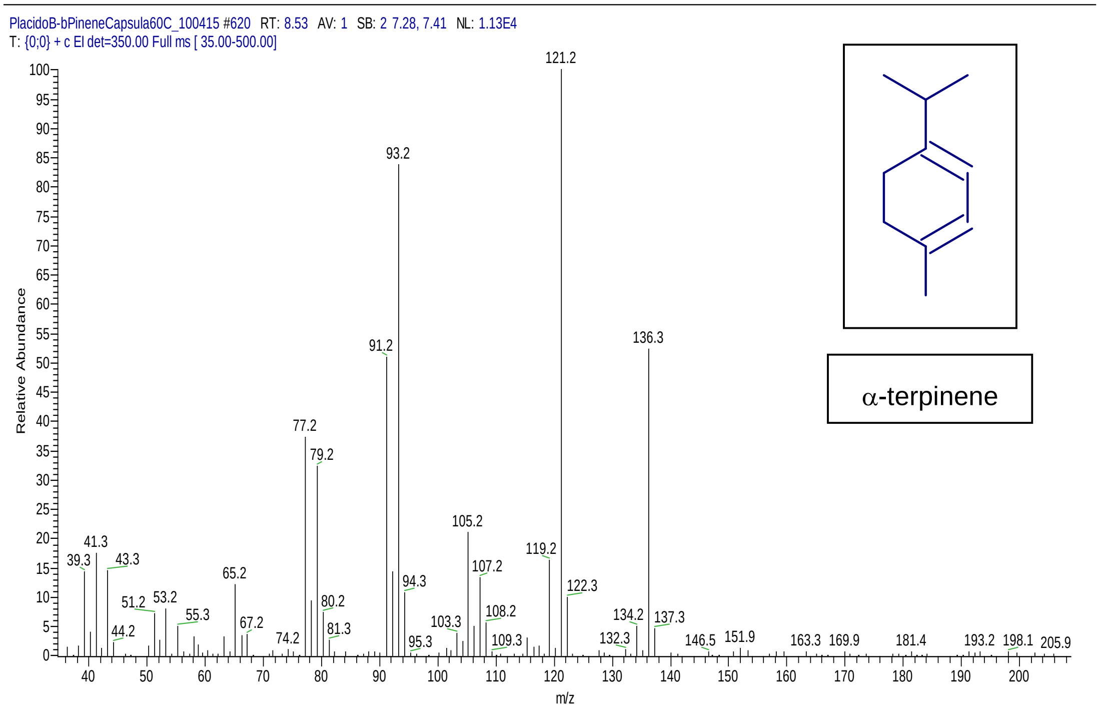

Figure S32: EIMS spectrum of α-terpinene

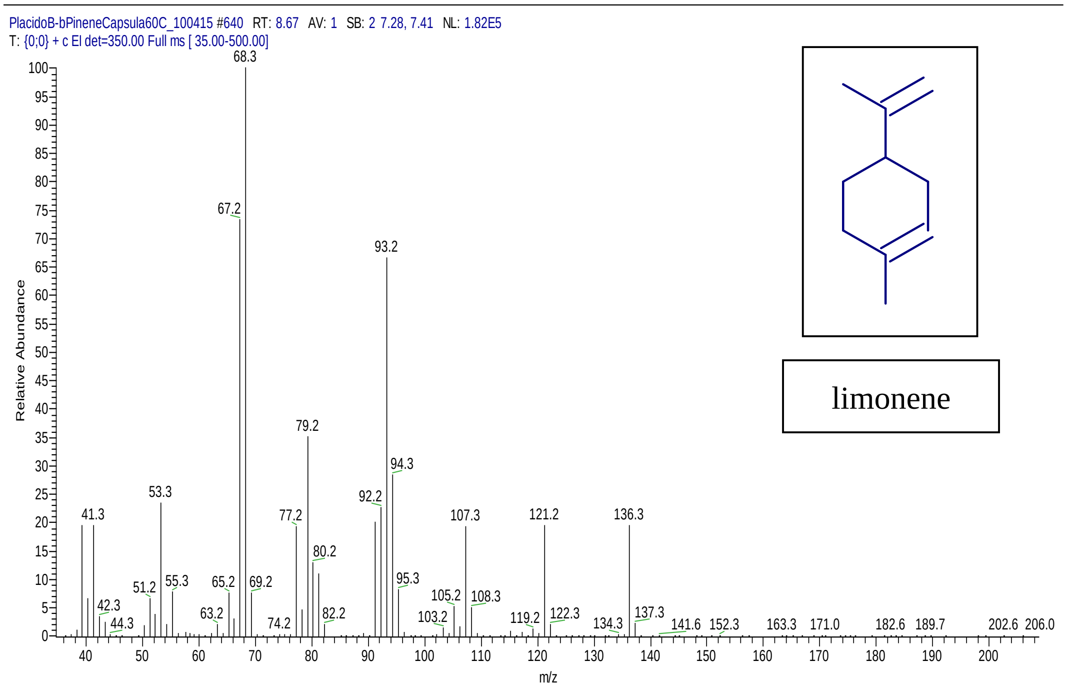

Figure S33: EIMS spectrum of limonene.

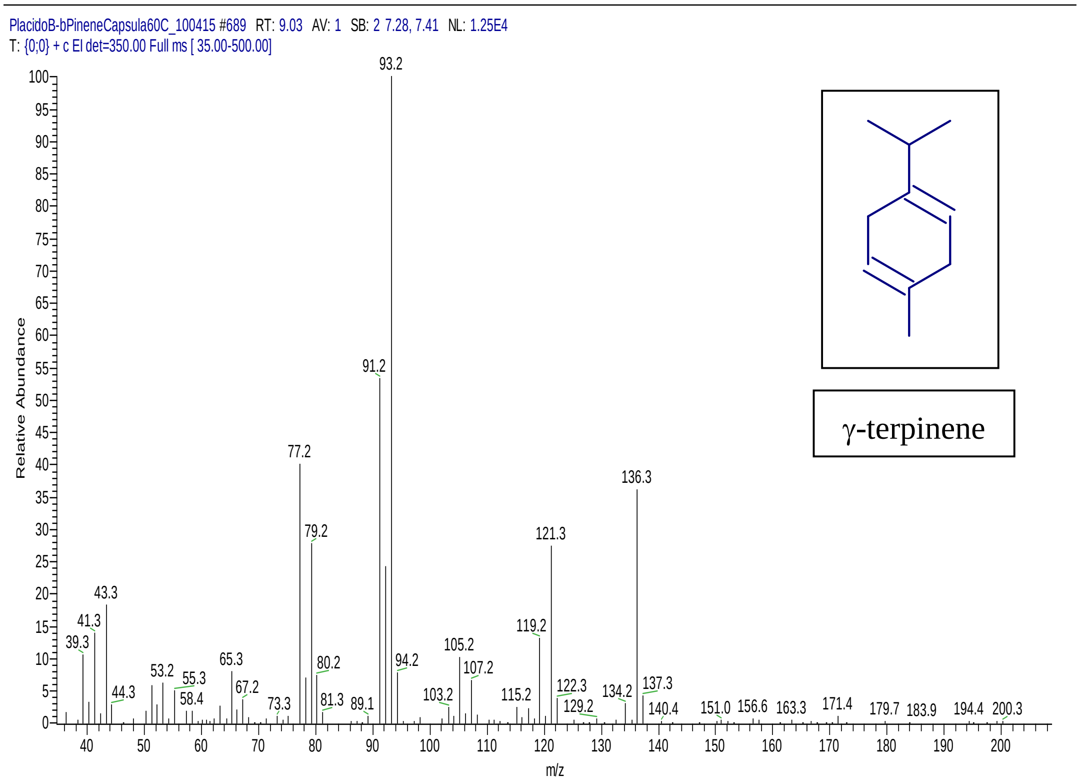

Figure S34: EIMS spectrum of γ-terpinene

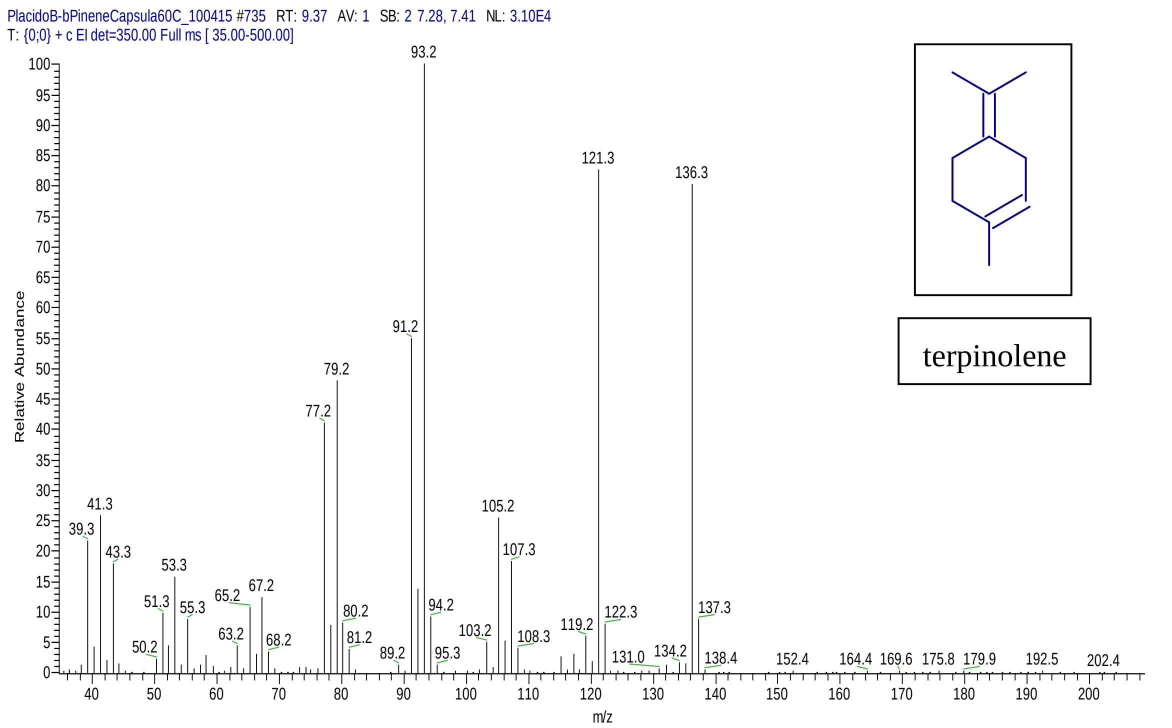

Figure S35: EIMS spectrum of terpinolene.

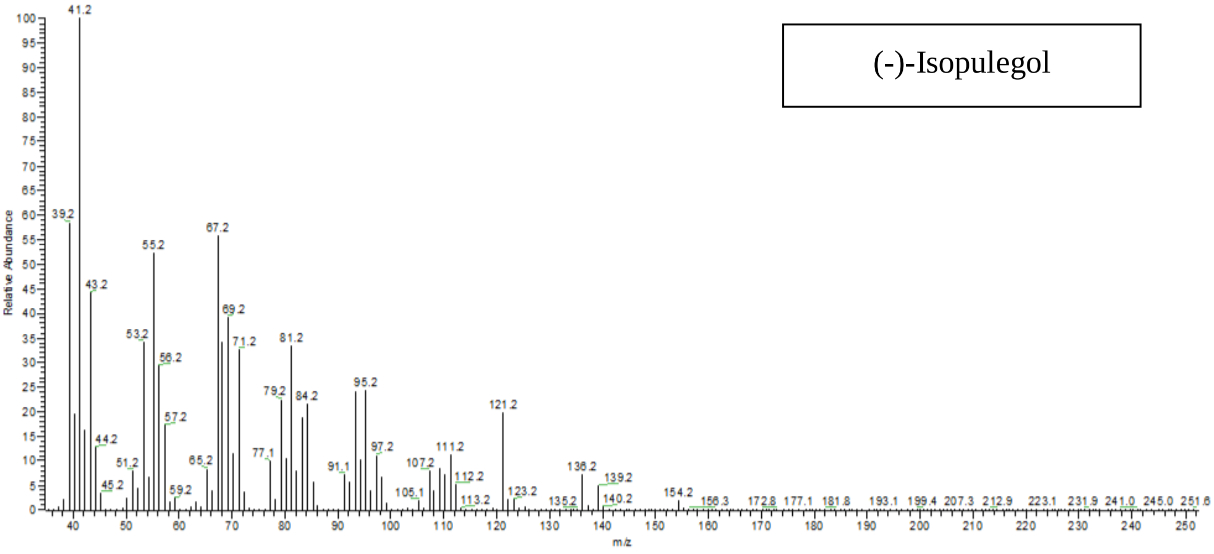

Figure S36: MSEI spectrum for (−)-isopulegol M.W. = 154

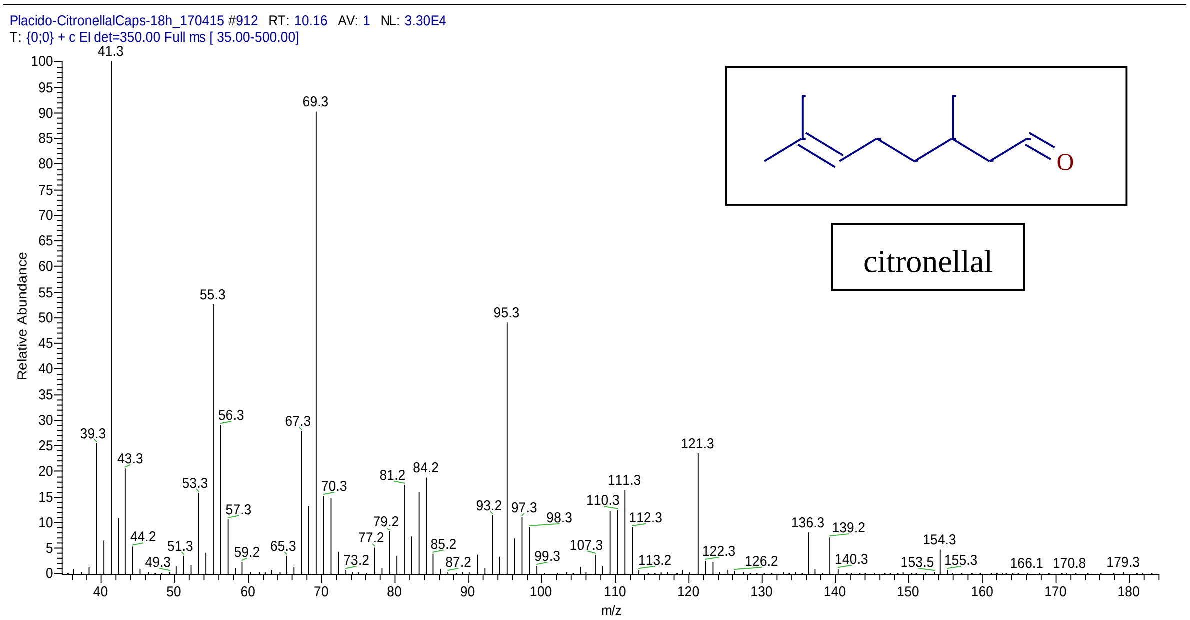

Figure S37: EIMS spectrum of citronellal.

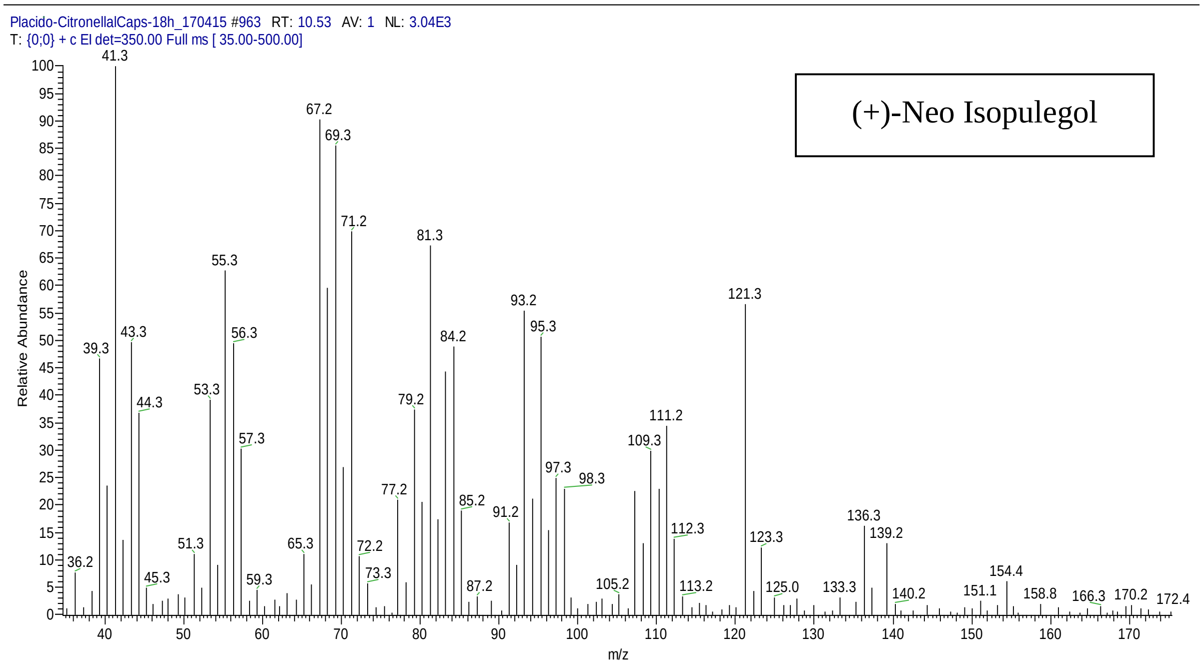

Figure S38: EIMS spectrum of neo-isopulegol.
